# Automated Infant Eye Tracking: A Systematic Historical Review

**DOI:** 10.1111/infa.70031

**Published:** 2025-07-22

**Authors:** Pär Nyström, Andrea Nesa Ziavras, Tekle Makashvili, Amelia Juslin, Venla Lehtonen, Amanda Riis, Gustaf Gredebäck

**Affiliations:** ^1^ Department of Psychology Uppsala University Uppsala Sweden

**Keywords:** eye tracking, infant, publishing trends, systematic review, WEIRD

## Abstract

Automated eye tracking has emerged as a powerful method in psychology, and has special benefits when studying infant populations. The field has developed much during the last decades, and while there are numerous reviews on methodological aspects and specific research topics, a general overview of the state and trends of the field has been lacking. That lack leaves the field unguided on several important aspects such as WEIRDness, statistical power and replication issues, unexploited areas of research, and the current status of the field as a whole. We here conducted a systematic review of the complete peer‐reviewed English literature on automated eye tracking with children during their first two years of life (793 articles), and extracted dates of publication, author and population geographic affiliation, keywords and sample sizes. The results show that automated eye tracking in infant research is increasingly used, and is accompanied by larger sample sizes, which together suggests improved accessibility. There is a focus on WEIRD populations, and a few broad research topics (methods, language and attention) and specific topics (autism, faces) are dominating the field. The current focus leaves many areas of research understudied, yielding a large potential for more infant eye tracking in the future.

## 1 Introduction

Eye tracking technology allow us to measure how infants perceive and interpret events in real time, what they choose to attend to, what they expect will happen in the future, and their real‐time surprise reactions to events that violate their expectations (Franchak et al. 2011; Gredebäck et al. 2010; Laeng et al. 2012). The technical advancement that made these methodological advancements possible is twofold; the high spatial (< 1 visual degree) and temporal resolution (50–1000 Hz) measures of infants gaze direction and pupil dilations on the one hand and the possibility to create automatic and more objective analysis protocols programmed in languages (such as R or MATLAB or through the software connected to the eye tracking system itself) that reduces the degree of subjectivity compared to past techniques (such as habituation or preferential looking techniques) (Aslin 2007). Automatic eye tracking (where an eye tracker has captured the gaze of infants and where automatic digital tools have been used to extract data) is a tool that has greatly impacted how infant research is conducted, and as a consequence thereof, has altered the type of questions asked in the field (Eckstein et al. 2017). However, the full and actual research output has not been explicitly addressed and quantified previously.

In this paper we present a systematic review of the field, focusing on automatic eye tracking with infants (0–2 years of age) and quantitative indicators such as the number of publications per year, distribution of age groups across studies, and the development of sample sizes over years. Further analysis report where authors and study populations in infant automatic eye tracking studies come from and what topics are being investigated.

Early studies of infant eye movements used observation to track infants' gaze, and observations and manual coding of infants vision has been reported in scientific outlets at least since the time of Darwin (Darwin 1877):

With respect to vision, ‐his eyes were fixed on a candle as early as the ninth day, and up to the 45th day nothing else seemed thus to fix them; but on the 49th day his attention was attracted by a bright‐coloured tassel, as was shown by his eyes becoming fixed and the movements of his arms ceasing. It was surprising how slowly he acquired the power of following with his eyes an object if swinging at all rapidly; for he could not do this well when seven and a half months old.

Darwin

While early reports pioneered the field, they typically had small sample sizes, where Darwin’s *n* = 1 marks the lower limit. They also had a methodological problem of subjectivity, since the gaze was judged by a human coder. To overcome this problem, the methods got increasingly sophisticated, using technological and methodological advancements such as filming, measuring, blinding of conditions, calculation of interrater reliability, etc. Unfortunately, all these improvements increased the time and resources needed for research. When automated eye tracking was introduced it was adopted as an often‐used research tool by many developmental researchers, it also allowed researchers to ask different, novel, research questions.

The first eye tracking devices were built at the late 1800s and early 1900s, but it was not until the 1920s that electrooculography (EOG) was developed that a tool suitable for infants was created (Young and Sheena 1975). EOG involves placing electrodes on the temple and forehead of infants whilst measuring the dipole of the eye as it moves in the horizontal plane, vertical tracking is also possible but more noisy due to the thickness of the skull bones (von Hofsten 2004). Another infant friendly method, the non‐invasive corneal reflection technique was developed in the 1930–40s (Buswell 1935). This technique calculates horizontal and vertical gaze (along with other measures such as pupil dilation) based on the reflection of near infra‐red light from the cornea and pupil. For a comprehensive history of eye tracking methods see (Wade and Tatler 2011). Currently, there are efforts to develop, and improve, other automatic eye tracking methods, such as web camera based eye tracking (Bánki et al. 2022; Steffan et al. 2024) and infant corneal‐reflection head‐mounted eye tracking (Franchak et al. 2011).

At the moment, there are many methods papers outlining various techniques for working with infant eye tracking data in typical populations (Gredebäck et al. 2010), in field studies (Leppänen et al. 2022), and in the context of neurodiversity (Falck‐Ytter et al. 2013). Analysis packages (Nyström et al. 2016; van Renswoude et al. 2018; Wass et al. 2013), dependent variables (e.g. Hepach 2024) and factors influencing the data (Hessels et al. 2015) are also well described. At the same time, review papers in specific fields suggest that automatic eye tracking is a standard tool in many infant research environments and that the technique is used to investigate topics as diverse as attention (Tu et al. 2022), face perception (Bastianello et al. 2022), object permanence (Gredebäck and von Hofsten 2007), and clinical populations such as autism (Falck‐Ytter 2024). These papers almost exclusively rely on corneal reflection techniques and it is fair to assume that this is prevailing eye tracking technique used at many developmental research labs working with infant populations.

What is currently missing from the field is a systematic review of all published papers relying on automatic eye tracking to assess infants’ psychological processes. In this systematic review, we assess the complete peer‐reviewed English literature on automated eye tracking with children during their first 2 years of life in order to describe the current state‐of‐art and report trends of the field. In addition to reporting descriptive information about number of studies over time and age groups assess, we target three overarching themes not previously assessed in the literature. For our purposes automatic eye tracking includes both the automatic detection and tracking of the infants’ eye gaze as they view stimuli and the use of programs to automatically determine the location of the eye gaze on the stimulus.

First, we ask where authors and study populations in infant automatic eye tracking studies come from, aiming to analyze the potential sampling bias in infant eye tracking (theme 1). This question is motivated by past reports that psychology research in general (Cheon et al. 2020; Henrich et al. 2010) and infancy work more specifically (Gredebäck et al. 2025; Singh et al. 2023, 2024) are largely based on WEIRD (White Educated Industrialized Rich and Democratic) populations. The degree to which infant automatic eye tracking research suffer from the same challenges or not is an open question. On the one hand, it is possible that the technique has been adopted by labs across the globe, without the need for experience with techniques such as habituation or preferential looking. On the other hand, it might turn out that the use of these tools has centered around traditional baby labs in WEIRD contexts, labs that have sufficient resources to purchase, develop and maintain competence and hardware over time.

Second, we ask what overarching topics are being assessed in infant research using automatic eye tracking (theme 2). This knowledge is needed to identify strong research areas, but also to identify topics that are left unexplored. As noted above, review papers of the past have targeted isolated topics. However, a complete view of the entire field is missing. A better understanding of the overarching themes beings investigated will help the field identify areas where a lot of research is being conducted and use this information to later identify areas that are left unexplored. As the number of papers expected to be included in this systematic review is high, we will focus on a quantitative analysis of key words rather than a quantitative analysis of each individual paper, a topic more suited for narrative or systematic reviews of individual sub‐fields (identified in the current paper and exemplified above).

Third, we ask what sample sizes are being used in the field, currently and historically. This knowledge is important as a benchmark for future studies and in order to further a critical discussion about appropriate power in infant automatic eye tracking studies. This data becomes especially important in the light of the recent replication crisis in psychology in general and infancy research specifically (Collaboration 2015; Frank et al. 2017; Kenward et al. 2017; van den Berg et al. 2022). Several studies have discussed the problem of small samples sizes in infant research more broadly (Oakes 2017; Rhemtulla and Bottesini 2022; Sen and Gredebäck 2024), however, a systematic review of samples sizes and changes in sample sizes over time in the field of automatic eye tracking is currently missing.

These themes are concretized as seven research questions addressed in this paper: Q1: When were the studies published and what is the trend over time? Q2: Where do the authors and study populations in infant automatic eye tracking studies come from? Q3: What are the most investigated topics? Q4: What are the most common publication outlets? Q5: Which automatic eye tracking methods are used? Q6: What infant ages are typically included in these studies? Q7: What is the average study size and how has this changed over time? By addressing these three themes and seven research questions in the same paper, it becomes possible to identify problematic practices and identify prosperous routes into the future. With this paper we hope to further promote ongoing debates about where the field currently stands and what needs to change in the future.

## 2 Material and Methods

This systematic review is based on the PRISMA method (Moher et al. 2009). The following criteria were established before the literature research and were used to determine the eligibility of the studies to be included in the review:

1. The study uses automated eye‐tracking methods and devices that did not require manual human coding. By automatic eye tracking we mean studies that use a technical devise that records the eye movement of infants (e.g. a camera, EOG) and where computer algorithms are used extract x and y coordinates of gaze and/or the diameter of the pupil and relate this to time‐locked stimulus events. Studies were included even if the eye tracking was used in conjunction with other methods, as long as there was at least one statistical analysis including eye tracking parameters.
2. The study measures eye‐gaze, pupil dilation, latency, or gaze velocity in response to external stimuli.
3. The study involves groups of human participants with a mean age younger than 2 years. After this age researcher can increasingly use language and other communicative methods instead of eye tracking, to tap into infants' development.
4. The study assesses infant behavior and/or psychological development, such as cognitive, socio‐emotional development.
5. The study is a quantitative study published in a peer‐reviewed journal, and written in the English language.

After an initial search and overview of the search records, the following exclusion criteria were defined to keep the included records within the scope of the study:

1. The study does not involve eye‐tracking methodologies or devices (such as manual coding, video coding, frame‐by‐frame coding, observation studies, EEG, or ERP studies).
2. The study does not involve external stimuli, and only measure physiological responses.
3. The study does not measure infant behavioral outcomes or psychological development.
4. The study only has participants older than 24 months, or only involve animal participants.
5. Systematic reviews, conference papers, theses, book chapters, editorials, and studies written in a language other than English were excluded.

The exclusion of papers that does not use automatic eye tracking and automatic coding is important as a boundary condition for this review. More specifically, infants looking is used in several other paradigms, such as habituation or preferential looking and these studies could in principle be described as eye tracking on a very rudimentary level (an experimenter assessing if an infant is attending to a particular stimulus or not based on video‐recordings of infant's face or in real time when observing an infant attending to stimuli). This is not the target of the current review. At the same time, there are studies that will have to be excluded when using this boundary condition that are extremely close to what has been done in the field of automatic eye tracking. Some early studies manually coded the detailed scan‐path of infants from video or from the output of an eye tracker (e.g. Kovack‐Lesh et al. 2014). It could be argued that these particular studies should have been included in this review. However, the focus on automatic eye‐tracking is a very clear boundary that will capture the absolute majority of studies and in particularly the work done in recent years where algorithms are easily accessible.

**FIGURE 1:**
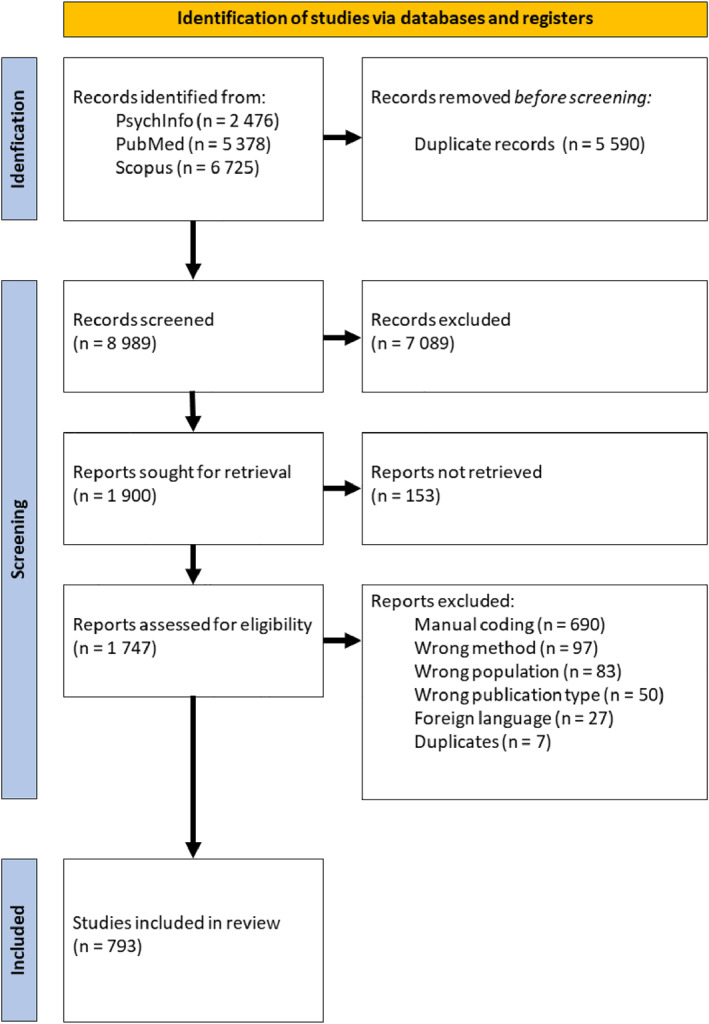
PRISMA Flow Diagram of studies identified for data extraction. The search was performed by external librarians, and the full search terms are found in the Supplementary Materials.

With the help of a librarian at the Uppsala University, a systematic search was conducted in three databases: PsycInfo, PubMed, and Scopus, with search terms including “eye‐tracking”, “gaze tracking”, “eye‐movement”, “oculomotor function”, “oculomotor dysfunction”, “oculomotor track*”, “infants gaze”, “infant”, “baby”, “month‐old*”, “newborn” and similar terms (see Supporting Information S1: Table SM1‐SM3 for full search strings in all three databases). The searches yielded over 14,000 articles as depicted in the PRISMA flow diagram (Figure 1). After removing duplicates, 8989 records were transferred to Rayyan software (a tool for systematic reviews that allow blind double coding of papers for inclusion) for initial screening (Ouzzani et al. 2016). Subsequently, a review team of five independent coders (the large number of coders is due to the vast numbers of screened papers) blindly screened the abstracts and titles of the articles to determine their eligibility for further inclusion and review. A minimum of two coders had to screen each article and conflicts were resolved by a third coder within the team. Of these 8989 articles, 1900 were sought for retrieval. Of these 1900, 1747 full text documents were retrieved and reviewed according to inclusion criteria. Of these, 793 articles were included in the final review (see Figure 1 for the different exclusion reasons). The 153 articles not retrieved was not accessible from the internet or locked behind a pay wall outside the university's publisher licenses and agreements.

The data extraction from the studies was conducted in several phases. In the first phase, the articles were assessed and coded by individual reviewers to manually extract the following data from each article:

1. Author affiliations: The first affiliation of all authors of each study, including country origin.
2. Methodology used: The type of the eye‐tracking device used in the study.
3. Study number: Number of sub‐studies/experiments included in each article.
4. Sample characteristics: Participant country origin, sample size, mean age, and information on sex/gender distribution.
5. Experimental conditions: Number of group conditions, and longitudinal study timepoints, if applicable.

If any of the reviewers was unsure of how to extract the required information from an article, the article was passed on to the second phase. During the second phase, all five individual reviewers discussed the passed‐on articles and extracted data according to consensus decisions. As a result, no articles were left unexamined, and the data extraction was completed for the entire set of included studies (i.e. consensus was reached for all articles, even in the passed‐on articles). In the third phase of the review process, the five individual reviewers checked the validity of the author affiliations extracted in the previous phases, and 177 articles were revisited to verify and correct author‐affiliation mismatches. This step ensured that the final dataset included accurate and complete author‐affiliation details for all included studies.

Curation, analysis and visualization was performed using the Time Studio framework (Nyström et al. 2016), using MATLAB r2022b. All data and analysis scripts are available at the Open Science Framework (https://osf.io/fsw5r/).

## 3 Results

In total, after data curation, 793 studies were included, and the results were extracted according to the seven research questions, Q1‐Q7.

### 3.1 Q1. When Were the Studies Published and What Is the Trend Over Time?

**FIGURE 2:**
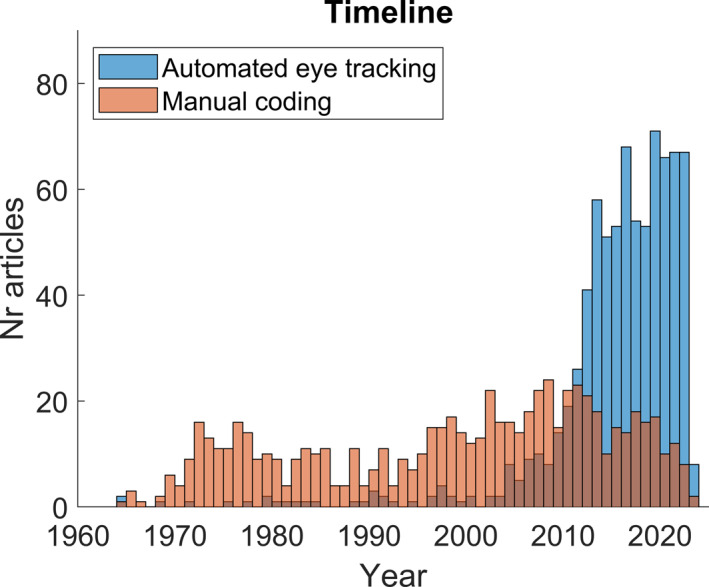
Number of both automatic (blue) and manually coded (orange) eye tracking studies published over time. The first published article in our data was in 1964. Corneal eye tracking studies appeared for the first time in 2004, and is now the most common eye tracking technique.

The records included for data extraction were published between 1964 and 2023 (last search was performed in August 2023). The oldest study from 1964 used EOG (Dayton et al. 1964), and the first corneal reflection study was published in 1975 (Mendelson and Haith 1975). However, as seen in Figure 2, articles were published very infrequently until about 2004, when there was a rapid increase. On average, 62 articles were published every year between 2018 and 2022, and the trend is still increasing.

As an exploratory analysis, we also investigated the trend over articles that had been excluded due to manual coding (“studies in which human observers recorded infants looking time, i.e. non‐automated eye tracking”). The pattern over time is very different, with a low but steady publication rate from the mid‐1960s with 5—15 articles published every year, and peaking at 20—25 articles around 2010. Interestingly, as the automated eye tracking rapidly increase, the number of manually coded articles decline, and the peak of the manually coded articles coincide with the maximum growth (steepest slope) of automated eye tracking.

### 3.2 Q2. Where do the Authors and Study Populations in Infant Automatic Eye Tracking Studies Come From?

**FIGURE 3:**
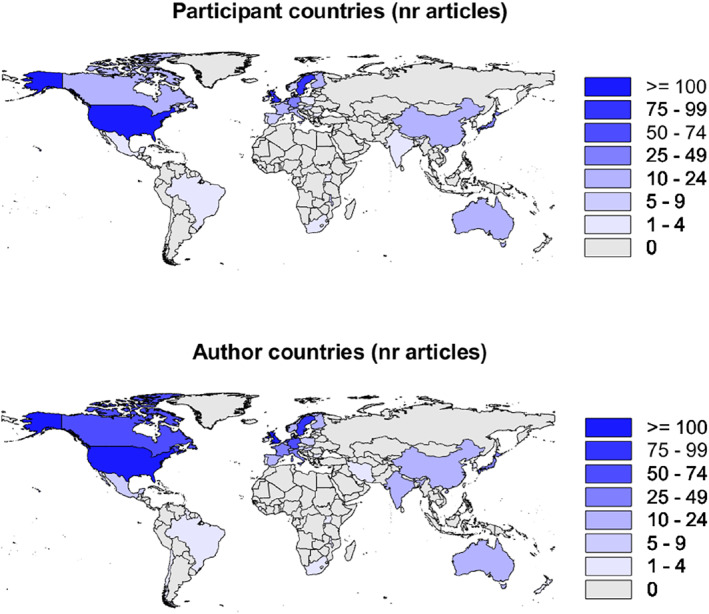
World map with participant countries (top) and author affiliation countries (bottom). Color shades represent the number of articles associated with each country. Shapes were provided by geoBoundaries (Runfola et al. 2020).

To investigate the origin of the articles we calculated how many articles had at least one author affiliated to each country (i.e. if an article had two or more authors from the same country, that article was only counted once for that particular country). A similar calculation was done for participant origins, and we found a strong correlation between the author affiliation count and the participant origin count for the 44 countries that had any data: Pearson r (42) = 0.986, *p* < 0.001, suggesting that our reliance on author affiliation might work as an index of where data was collected, even when this was not explicitly mentioned. Where the origin of participants was not stated (*n* = 218, i.e. 27.2%), it was assumed to be the same country as the first author, and frequency world map and rank list was calculated (Figure 3 and Table 1). This will not be a perfect indicator on each individual instance, but the assumption here is that authors that make the additional effort to go to a different country to collect data will most likely see this as important and integrate this information in the paper (describing the sample or context of the data collection). This decision is also informed by what we perceive as a lack of attention to this issue in the past, where WEIRD researchers have studied participants in WEIRD contexts without reflecting on the lack of representativeness and importance of this limitation, something that might result in a failure to report the geographical or ethnical origin of participants.

**TABLE 1:** Countries with most publications linked to affiliation countries and study population countries.

Rank	Author country	No. articles	Participant country	No. articles
#1	U.S.A.	495	U.S.A.	278
#2	U.K.	139	U.K.	100
#3	Sweden	89	Sweden	79
#4	Germany	66	Germany	57
#5	Netherlands	59	Netherlands	52
#6	Canada	51	Japan	48
#7	Japan	48	Italy	24
#8	France	39	Canada	22
#9	Italy	35	France	21
#10	Finland	23	Finland	19
#11	Australia	21	China	13
#12	Switzerland	16	Australia	11
#13	India	14	Switzerland	9
#14	China	14	Spain	9
#15	Norway	13	Belgium	7
#16	Spain	12	Poland	6
#17	Hungary	9	Hungary	6
#18	Austria	7	Norway	6
#19	Poland	7	Singapore	5
#20	Belgium	7	Malawi	5

The most represented countries were U.S.A., U.K, and Sweden, with other European countries and Japan within the top ten.

### 3.3 Q3. What Are the Most Investigated Topics?

A detailed semantic analysis of the article contents was not possible due to resource demands, but all articles except one had keyword metadata provided by the journal that could be extracted and analyzed. These keywords were automatically extracted from the librarian search procedure's data file (a “.ris” file containing all raw information, such as article title, author names, journal, abstract, and keywords). The mean number of keywords was 14.7 (std = 6.4), and only 42 studies provided 5 or less keywords, which suggests that authors and journals generally provide meta‐data to a large extent (since we extracted the keywords from the librarian references, it was impossible to separate authors' keywords and journals meta‐tagging keywords). Word counts of the abstract content was also performed, but because the results from the abstract was very much in line with the keywords analysis (though cluttered with irrelevant words such as “and”, “or”, and other syntactical words), only the keywords analysis is presented here. A full list of key words, the raw data for our analysis, is available in the supplementary materials. This list was analyzed by two of the authors, by selecting all key words with a count ≥ 10 and with connotations to psychological or methodological constructs for a thematic analysis. In the thematical analysis the key words were categorized by the authors' judgments into conceptual research topics in two iterations, by assessing each keyword one by one and deciding whether the keyword could be assimilated into an existing topic or whether a new topic was needed. If a key word was assessed differently by the two coders it was resolved by discussing and if disagreement remained, by asking a third author. An overarching topic called “Perception/cognition” was split into two topics, “Perception” and “Cognition”, at the second iteration of the thematical analysis, yielding a total of eleven topics shown in Table 2. We acknowledge that some keywords may fit in multiple topics, due to overlap between topics or the hierarchal structure of topics, but each keyword was only included in one category to avoid inflating some keyword counts. Because the selection of topics and categorization of keywords was primarily based on the two authors' judgments the actual keywords and their counts are also presented for full transparency and to allow for re‐interpretation. Overall, the analysis reveals a strong focus on “methods”, followed by “language”, “attention”, and “clinical”. Not surprisingly, broad keywords such as “attention” (n = 277), “language” (n = 136) and “learning” (n = 101) had large counts, but also more specific keywords such as “autism” (n = 156) and “face” (n = 111) appeared as important to the field.

**TABLE 2:** The most common research topics and keywords.

Rank	No. occurrences	Topic	Keywords
#1	620	Methods	**[Eye] fixation (186), photic stimulation (122)**, [smooth] pursuit (56), saccades (51), reaction time (45), pupil [lometry] (43), anticipation (32), visual tracking (30), visual fields (13), prediction (10)
#2	376	Language	**Language [development] (136)**, speech [perception] (85), vocabulary (39), verbal learning (22), semantics (20), word learning (19), language acquisition (18), phonetics (15), multilingualism (12), linguistics (10)
#3	355	Attention	**Attention (277)**, visual attention (50), attentional bias (17), selective attention (11)
#4	315	Clinical	**Autism [autism spectrum disorder, autistic disorder, autisms] (156)**, physiopathology (53), diagnosis (42), child development disorders (36), premature (28)
#5	245	Social	Interpersonal relations (37), communication (33), social behavior (33), social perception (32), social cognition (32), goals (19), gaze following (16), gestures (14), mother‐child relations (14), imitative behavior (11), joint attention (10)
#6	237	Perception	Pattern recognition (77), motion perception (45), orientation (45), discrimination (15), perception (15), acoustic stimulation (14), space perception (14), auditory perception (12)
#7	200	Faces	**Face [perception, processing] (111)**, facial expression (45), facial recognition (31), mouth (13)
#8	168	Emotion	Emotion [s] (62), fear (29), temperament (19), happiness (18), arousal (15), anxiety (13), anger (12)
#9	154	Cognition	Cognition [cognitive development] (91), concept formation (28), intention (13), executive function (12), motivation (10)
#10	121	Learning	**Learning (101),** association learning (10), habituation (10)
#11	90	Memory	Memory (61), recognition (29)

*Note:* Merged keywords are marked with brackets [ ], and keywords with > 100 occurrences are marked with bold font.

### 3.4 Q4. What Are the Most Common Publication Outlets?

The publication outlet is also a marker of research topics, since scientific journals have specific scopes and audiences. To investigate which journals publish most articles using automated eye tracking, we created a rank table with the journal names. As seen in Table 3, journals focusing on infancy and development typically have high ranking, which is not surprising considering the search terms used in our literature search. However, more general journals are also well represented among the top publishing journals, with Cognition, Frontiers in Psychology, and PLOS One among the top ten. This shows that infant eye tracking is not only confined to highly targeted journals, but also carry potential for impact on broader audiences.

**TABLE 3:** The ten most common scientific publication outlets.

Rank	No. occurences	Journal
#1	59	Infancy
#2	52	Developmental Science
#3	50	Infant Behavior and Development
#4	48	Journal of Experimental Child Psychology
#5	36	Developmental Psychology
#6	34	Cognition
#7	34	Frontiers in Psychology
#8	32	Child Development
#9	32	PLOS One
#10	20	Developmental Psychobiology

### 3.5 Q5. Which Automatic Eye Tracking Methods Are Used?

The analysis of eye tracking methods showed that a vast majority of studies used corneal reflection studies: 659 studies used remote corneal reflection, 26 additional studies used corneal‐reflection head‐mounted eye tracking, 24 studies used EOG, 3 studies used automated video analysis, and 41 studies used other or unspecified methods. A separate analysis of the corneal reflection studies' publication years showed that only 10 studies was published sporadically between 1975 and 2003. After 2004, when 5 studies were published in the same year, the rates increased rapidly, and during the 5‐year interval 2018–2023, the average number of corneal reflection studies were 56.8 publications per year.

### 3.6 Q6. What Infant Ages Are Typically Included in These Studies?

**FIGURE 4:**
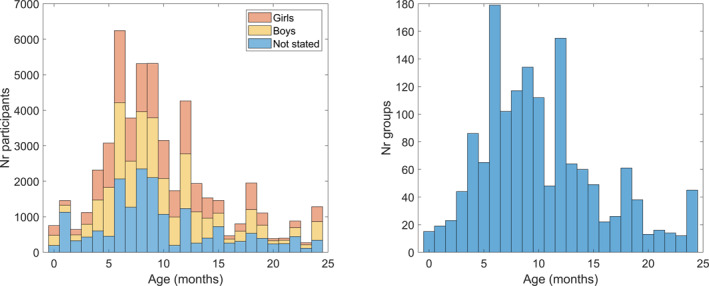
Distribution of the number of participants in total and for boys, girls, and not stated (left panel) and how they were stratified into age groups (right panel). The ratio girls/boys varied between 0.586 and 1.126, and the ratio (girls + boys)/not stated varied between 0.126 and 3.418, indicating considerable variation in reporting.

To investigate which age groups have been studied we extracted data for individual study groups, as presented by the articles. In this analysis, participants in longitudinal studies contributed to the sample size at every visit (i.e. a group of 10 infants coming to the lab 3 times would get a sample size of 30). The data included the number of participants of each study group, how many participants were girls and boys, and how many participants did not have any sex stated (Figure 4, left panel). To get an overview of how studies typically select their age groups we also counted the total number of groups in the studies (Figure 4, right panel).

The results show an irregular distribution of age groups, with local peaks at 0, 4, 6, 9, and 12 months of age. Overall, the age range 4—10 months is well represented, with a global maximum at 6 months, and few studies assessing 2‐month‐olds and infants older than 14 months. There was no significant difference between the number of boys and girls.

### 3.7 Q7. What Is the Average Study Size and How has This Changed Over Time?

**FIGURE 5:**
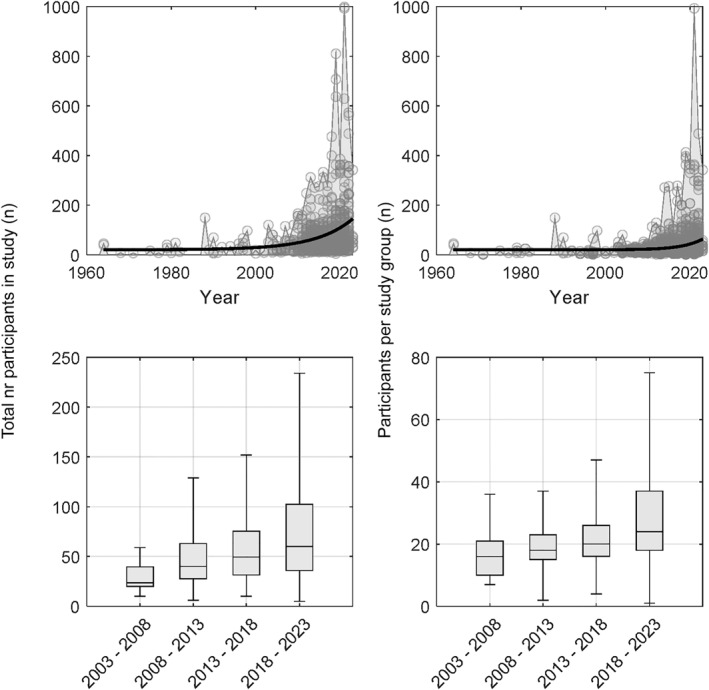
Scatterplot of total participant count (top left) and study group sizes (top right) across years. An exponential model was fitted and plotted in black thick line, and the max values are shown as a light blue or gray area. Bottom panels show box‐and‐whisker plots for 5‐year‐intervals, with outliers removed (not plotted for scaling benefits). Whiskers show min and max values, and box covers the 25:th to 75:th percentiles with the median marked with a black line.

As seen in Q1, more and more studies are using automated eye tracking to study infants gaze behaviors, and it is possible that also sample sizes have been changing. To address this question, we used the study year as x values and the total participant count from Q6 as y values, and fitted a one‐term exponential model (Figure 5, top left panel), which provided a better fit than polynomial models according to the adjusted *R* ^2^ = 0.082. Because of the longitudinal design in some studies, and that the number of study groups varied between studies, we also counted the number of participants in each study group (Figure 5, top right panel), *R* ^2^ = 0.053.

The exponential curve fit showed a clear increase in sample sizes used, both for studies and for study groups, with marked growth acceleration between 2010—2023. The average total study sample size during 2018—2022 was 114.8 (median = 69), and the average group sample size was 45 (median = 26). For the group sample sizes, there is one noteworthy extreme datapoint representing the largest group sample size (Chang et al. 2021), *n* = 993, with more than twice as many participants as the second largest group sample size, *n* = 488.

To extract current study norms and trends over time, we pooled studies within 5‐year intervals and calculated the min, max, quartiles, and the median for both the total study sizes (Figure 5, bottom left), and for group sizes (Figure 5, bottom right). These descriptive are found in Table 4.

**TABLE 4:** Descriptive statistics of total study sizes and group sizes.

Total participants in study				
Years	2003–2008	2008–2013	2013–2018	2018–2023
Median	23.5	40.0	49.5	60.0
Min	10.0	6.0	10.0	5.0
Max	59.0	129.0	152.0	234.0
25 percentile	20.0	27.5	31.5	36.0
75 percentile	39.5	63.0	75.5	102.5

Participants per study group				
Years	2003–2008	2008–2013	2013–2018	2018–2023
Median	16.0	18.0	20.0	24.0
Min	7.0	2.0	4.0	1.0
Max	36.0	37.0	47.0	75.0
25 percentile	10.0	15.0	16.0	18.0
75 percentile	21.0	23.0	26.0	37.0

## 4 Discussion

The results show several trends in line with high popularity and versatility: after the turn of the millennia automated eye tracking has increased enormously in comparison to manually coded eye tracking; at least in some countries of the world. During the same time sample sizes have also increased and a wide variety of topics are addressed, especially in the ages between 4 and 12 months.

Despite the difficulty of interpreting and extrapolating trends into the future, it is worth discussing a few points in more detail. For example, there was an increasing trend in manually coded studies until the rapid increase in automated eye tracking around 2004—2010. After that, the number of manually coded studies have steadily decreased, giving an impression of competition between the methods where automated eye tracking has not only replaced manual coding, but also increased the interest in infant eye tracking. Clearly, it is not possible from this data to know if there is a causal relationship between the trends or what the reasons are (there could have been an evenly large increase in manually coded studies if automated eye tracking had not entered the market), but it is tempting to interpret the coinciding trend changes as an effect of accessibility. Around that time, several automated eye tracking companies started selling research grade eye trackers with user friendly software that reduced the resources needed to do infant studies, which could explain the rapid increase in published articles.

Regarding to the geographic distribution of studies and representation of countries (theme 1), it is obvious that most studies have been performed in white, educated, industrialized, rich, and democratic countries, that is WEIRD countries (Henrich et al. 2010). The skew toward WEIRD publications in our data, and scarcity of articles from the Global South, is very much in line with a recent large‐scale review of developmental studies overall (Singh et al. 2023). In Singh et al.’s review, a wide range of articles (*N* = 1682) published between 2011 and 2022 in four major infant research journals, there was a variety of methods used and no specific mentioning of eye tracking. Our findings complement theirs by providing data from a specific method, longer time interval (1964—2023), and the inclusion of all articles regardless of journal. Restricting the study contexts (regardless if it is WEIRD or non‐WEIRD) reduce the possibility to generalize and may prove scientifically costly, as WEIRD participants have been shown to provide extreme responses in many experimental tasks and risk biasing our interpretations of human behavior. It is therefore an important future prospect for the field to self‐examine inequities, diversity study contexts by building international collaborations, and acknowledging limitations when generalizations are done from limited samples to the global population.

When it comes to research topics (theme 2), interpretation of the results is more difficult. The frequency of words is not a direct proxy of semantics or importance. Some keywords are linked to the methods or the overarching topic rather than the research question, and some key words may therefore get an inflated word count. For example, “attention”, the most common psychological construct in our data, can refer both to research questions regarding attention and the fact that eye tracking is tightly linked to visual attention. Another problem is that topics may be fragmented by different uses of key words, and it is possible that the key word “visual attention” has the same meaning as some of the “attention” studies. To resolve these issues a full read‐through of all papers had to be done, which was not possible with the resources for the current study. With these caveats in mind, it is still possible to infer that a large portion of the studies highlight methodological aspects. For psychological constructs, attention, language, autism, and faces, exemplify topics that the field has gravitated toward and added mass to. It is notable that there seem to be a special interest for “autism” (*n* = 156) and “faces” (*n* = 111), which have counts on par with broader topics such as “Emotion” (*n* = 168) and “Cognition” (*n* = 154). Similar to the geographic and cultural representation of studies, we find that the field has not yet fully utilized the potential of diversification. Taken together, with the accessibility of the method and the possibility to use small and very portable eye trackers in mobile setups, we see no obvious obstacles for unlocking this potential, regardless whether it is fueled by cross‐topic, cross‐cultural, interdisciplinary research, or any combination of these. For example, a recent study (Gredebäck et al. 2025) combined different countries (> 800 infants from Bhutan, Sweden, Uganda and Zimbabwe) with different environmental challenges (through maternal mental health, exposure to climate adversities such as draught or starvation, and extreme war experiences) and showed remarkable robustness in infants' gaze following (highlighting the robust null effects, as shown by Bayesian statistics). This example is related to United Nations sustainable development goals (Transforming Our World: The 2030 Agenda for Sustainable Development, 2015), which we believe is an important platform that can be used for generating and guiding research prospects. Further, when video‐based eye tracking becomes more widely used, the possibilities to do eye tracking remotely will further enhance the possibilities for diversifying infant eye tracking studies. With such a huge space for exploration and unmet needs, we encourage both experienced researchers and novel scientists to enter new areas, and start solving the diversification problem.

The participant ages showed an irregular distribution with notable peaks at 4, 6, 9, and 12 months of age, and fewer participants at 2, 15 and 16 months of age. Very few studies assessed ages above 20 months. The focus on the 4—14 months age range is likely due to both practical and theoretical reasons. Practical reasons include that infants become able to sit stable for extended periods at this age, which makes the testing situation easier to set up, and they can most often keep their attention on the screen. Younger participant needs more support to be able to watch the screen and have shorter attention spans, and the very youngest may have eye shapes that makes eye tracking data noisier (Wass et al. 2014). Older participants may find repetitive stimuli (which is often needed to extract systematic gaze responses) boring, or find exploring the room or social interactions more interesting; all of which are factors that inflate noise and/or missing data. The problem of noisy and missing data is important, and we will discuss it in more detail later. The theoretical reasons for the jagged age distributions include the rapid expansion of the infants' behavior repertoire after the first couple of months, which reflects that many perceptual and cognitive circuits are maturing to a functional form that are of theoretical importance. For example, while control systems for fixations and saccades are functional already at birth, smooth pursuit develops high speed visual tracking at around 3—5 months of age (Roucoux et al. 1983; Von Hofsten and Rosander 1997). The peaks and particular focus at 4, 6, and 12 months may be due to conventions within the field: in order to relate to previous research, it is important to control for age by studying the same age groups. Over all, the research questions should guide the choice of age groups to study, and our results does give some support that it is the current practice in the field.

Study sizes have increased over time, with a sharp onset between 2004—2010, showing that automated eye tracking has become more and more accessible and is engaging more participants than ever. Large sample sizes are generally good, as they minimize the risk of type I and II errors, and avoid undermining the internal and external validity. It also makes it possible to create more complex statistical models without overfitting the model to the data (and again risking over‐generalization). However, it should also be noted that very large sample sizes may detect significant differences even when the effect size is so small it becomes irrelevant for the research question. Too large sample sizes also waste resources, and it is therefore important to consciously estimate the pros and cons of different sample sizes and calculate the statistical power (Faber and Fonseca 2014). With that said, we conclude that most studies currently use 18—37 participants per study group currently (median = 24), and researchers designing new studies should preferably not plan for lower numbers.

Assessing the study and group sizes also allows for speculations regarding problematic practices in science and reproducibility and replicability issues (theme 3). The increasing study sample sizes show that more resources are used for infant eye tracking, but it does not say whether the statistical analyses are adequately powered. For example, a study with 100 participants could potentially have 10 sub‐experiments with only 10 participants in each experiment, which could be further stratified into 2 groups with 5 infants in each group, leading to very low power for individual analyses despite having a large study sample size. However, because both the study sizes and the group sizes are increasing, the risk of salami‐slicing and low powered analyses should be steadily decreasing. Combining this with the interpretation of theme 1 and 2 (i.e. rather homogenous populations and topics), it is likely that these research fields are first to be saturated and reach consensus. In the current study, we did not extract *p*‐values or effect sizes due to resource constrains, which limits the possibility to directly assess reproducibility, statistical power, eventual publication biases, and other important aspects of the study statistics in detail. It was also unclear whether the included studies were original or replication attempts, and consequently the analysis of trends in sample size can only act as an indirect measure of changes in the ability to replicate findings over time. Extracting that type of data is a remaining important question for future studies (Collaboration 2015; Frank et al. 2017).

Regarding the group sizes, the largest group had 993 participants (Chang et al. 2021), which is a noteworthy high group size. On closer inspection, the method used to achieve the large sample size also stand out. Chang et al. use automated video analysis, which utilizes machine learning to extract gaze behaviors from video input. This is not yet a common method: in our results from Q5 only 3 studies used automated video analysis, while 659 studies used remote corneal reflection, 26 studies used head‐mounted corneal reflection eye tracking, 24 studies used EOG, and 41 studies used other or unspecified methods. However, since the last search of this review in August 2023, a few more studies using automated video analysis have been published, and using deep learning and artificial intelligence to improve video analysis is an active research field. Using video recordings to estimate gaze behaviors unlocks new potential for automated eye tracking. For example, it becomes possible to perform data collection in people's homes without test leaders (Shic et al. 2023), and the recordings are easy to perform and very cost‐effective, which could help mitigate the WEIRD‐ness of the field. It could also help reduce the amount of missing data, although this has yet to be empirically proven with infants. Such advantages makes automated video analysis an attractive alternative to corneal reflection techniques, but it should be noted that it comes with a cost of lower accuracy and precision (Valtakari et al. 2023), limiting the design of stimuli and possible research questions. Again, the research question should guide the choice, and the coming years will show whether automated video analysis fulfills its potential and which research questions it will answer.

For research question Q5, the extraction of eye tracking methods showed a clear majority of corneal reflection studies (*n* = 659). As mentioned in the introduction, this method has a number of benefits: it is non‐invasive, suitable for all ages, accessible and cost‐effective. It has high temporal and spatial accuracy and precision, and it provides additional pupillometric information. However, there are also issues that require careful consideration when interpreting the data. First and foremost, infant eye tracking is associated with lower quality and more missing data compared to adults' (Hessels et al. 2015; Wass et al. 2014), and the younger the infants the worse the quality gets. The quality is highly dependent on the initial calibration (Schlegelmilch and Wertz 2019) and can decline during sessions (Wass et al. 2014). Poor quality can impact several key measures of eye tracking, and may introduce biases in preferential looking, slower reaction time latencies and shorter dwell times in areas of interest (Wass et al. 2014). This raises concerns about the internal validity of the method, but can also impact the external and ecological validity. Previous studies has shown discrepancies between gaze behaviors for stimuli presented on screens versus in real life (Diener et al. 2008; Wass 2014), and it is not always straightforward to link infants gaze behaviors to the assumed underlying cognitive mechanisms (Aslin 2007; Aslin and McMurray 2004). With more and more studies performed, each assessing more and more infants, there is an increased responsibility to acknowledge what we can and cannot infer from infant eye tracking data. Remote eye tracking, video‐based eye tracking, and head‐mounted eye tracking each offer distinct advantages and limitations. Remote corneal‐reflection eye tracking provides high spatial and temporal resolution, making it well‐suited for controlled experimental settings. It is non‐invasive and effective for capturing both gaze and pupil variables, but its reliance on calibration and the infant's ability to remain still can result in data loss, particularly in younger age groups. Video‐based eye tracking enhances accessibility by allowing data collection in naturalistic settings, including home environments. This method reduces the need for expensive hardware, but its accuracy is lower, and deep learning algorithms are required to extract meaningful gaze data. Head‐mounted eye tracking offers a unique advantage by capturing gaze from a first‐person perspective, facilitating studies on active exploration and real‐world interactions, but is constrained by the weight and fit of the device, potential data contamination due to head and body movements, and the resources required to analyze the data. However, methodological development in steady, and researchers should carefully monitor the full range of options and their current capabilities, and use what best suits their research question.

Before wrapping up, we want to synthesize two important implications from our results, as potential predictions for the future. First, by combining the exponential increase in sample sizes with the increasing number of publications each year, we believe that resources put into infant eye tracking is far from reaching an asymptote. Although the number of publications per year seem to flatten out, we think that there are many underexploited areas of research that could reignite the field. Secondly, referring back to the introduction, when new technology becomes accessible it is likely that researchers adapt it. In our geographical analysis we found many understudied regions and populations (i.e. non‐WEIRD), which might reflect that eye tracking technology simply is not accessible. This can change rapidly if video‐based eye tracking is cross‐culturally validated and can be used remotely over the internet. When this happens, we predict a similar increase in publications as when corneal eye tracking was introduced around 2004.

As always, there are several caveats that deserves being mentioned. First of all, there are many potential other analyses that could have been done on this data, for example analysis of drop‐outs, a formal meta‐analysis of included papers, or further in‐depth qualitative analysis of topics or sub‐topics. All of these would add valuable information to the field. We have selected a narrow range of analysis for two reasons. First of all, the number of papers included in this systematic review is staggering. With close to 800 included studies the work required for in‐depth topic analysis as one example would be enormous. Secondly, the diversity in reporting practices in combination with the vast number of paradigms, dependent variables and ages makes this dataset sub‐optimal for meta‐analytic approaches. Some of the potential analysis (meta‐analysis, qualitative analysis of topics) might be better served by targeted reviews focusing on a single area such as attention or face perception where the full range of paradigms and dependent variables can be explored. In addition, as this is a historical analysis of all automatic eye‐tracking there are shifts in conventions of what to report. When going through the volume of papers we get the clear impression of a field that is maturing with more standardized reporting of calibration quality, sample characteristics, and study details as years go by. From our perspective, this overarching paper is not the end, but the first holistic description of the field, hopefully complemented in the years to come with additional systematic reviews of sub‐fields, by us and others.

## 5 Conclusions

In conclusion, this study takes a systematic review approach that provide quantitative data confirming that eye tracking for infant research has developed tremendously during the last decades. On the positive notes, we find that the number of studies and number of participants has increased. On the negative notes, it appears that the field is rather restricted to WEIRD contexts and that few specialized research topics dominate the field. For the future, the neglected areas hold potential to unlock an even higher popularity, perhaps facilitated by advances in automated video analysis, and we urge actors in the field to explore these spaces and encourage others to do the same. A lot has happened since Darwin's anecdotal eye tracking report in 1877, and automated eye tracking in infant research is in a good position to continue its rapid evolution.

## Author Contributions

**Pär Nyström:** conceptualization, data curation, formal analysis, software, supervision, visualization, writing – original draft, writing – review and editing. **Andrea Nesa Ziavras:** conceptualization, data curation, formal analysis, writing – original draft. **Tekle Makashvili:** conceptualization, data curation, formal analysis, writing – original draft. **Amelia Juslin:** conceptualization, data curation, formal analysis, writing – original draft. **Venla Lehtonen:** conceptualization, data curation, formal analysis, writing – original draft. **Amanda Riis:** conceptualization, data curation, formal analysis, writing – original draft. **Gustaf Gredeback:** conceptualization, funding acquisition, writing – review and editing.

## Ethics Statement

The authors have nothing to report.

## Consent

The authors have nothing to report.

## Conflicts of Interest

The authors declare no conflicts of interest.

## Supporting information

Supporting Information S1

## Data Availability

Materials and analysis code are available at the Open Science Framework (https://osf.io/fsw5r/).
