# Supplementary material for: Automated Infant Eye Tracking: A Systematic Historical Review
Source: Infancy. 2025 Jul 22;30(4):e70031. doi: 10.1111/infa.70031 (PMC12284131; doi:10.1111/infa.70031)
Supplement: Supplementary file 1 — Supporting Information S1 [file INFA-30-0-s001.docx]

Supplementary materials

To article “*The advent of automated infant eye tracking: a systematic review*”

Authors: Nyström, P., et al.

**Table SM1.** *Literature search in PsycInfo 2023-08-22.*

| Search number | Search term | Number references |
| --- | --- | --- |
|  | Eye-tracking |  |
| 1 | (DE "Eye Fixation")  OR  (DE "Eye Movements")  OR  (DE "Visual Tracking") | 31 719 |
| 2 | "eye follow*" OR eyemovement* OR eye-movement* OR eyetracker OR "eye tracker" OR Eyetracking*  OR eye-tracking* OR "gaze follow*" OR "gaze instability" OR "gaze stability" OR "gaze stabilization" OR "gaze tracked" OR gazetracking OR "gaze tracking" OR "infant gaze" OR "infants gaze" OR "ocular mot*" OR "oculomotor function*" OR "oculomotor dysfunction*" OR "oculomotor track*" OR "visual tracking*" [Title/abstract] | 32 109 |
| 3 | 1 OR 2 | 45 011 |
|  | Infant |  |
| 4 | DE "Infant Development" | 22 160 |
| 5 | baby* OR babies OR infancy OR infant* OR month-old* OR months-old* OR "months of age" OR newborn* OR "new born" OR "new borns" OR "newly born" OR neonat*  [Title/abstract] | 138 004 |
| 6 | 4 OR 5 | 139 343 |
| 7 | 3 AND 6 | 2 522 |

**Table SM2.** *Literature search in PubMed 2023-08-21.*

| Search number | Search term | Number references |
| --- | --- | --- |
|  | Eye-tracking |  |
| 1 | Eye Movement Measurements [MeSH] OR Eye Movements [MeSH] | 59 422 |
| 2 | "eye follow*" OR eyemovement* OR eye-movement* OR eyetracker OR "eye tracker" OR Eyetracking*  OR eye-tracking* OR "gaze follow*" OR "gaze instability" OR "gaze stability" OR "gaze stabilization" OR "gaze tracked" OR gazetracking OR "gaze tracking" OR "infant gaze" OR "infants gaze" OR "ocular mot*" OR "oculomotor function*" OR "oculomotor dysfunction*" OR "oculomotor track*" OR "visual tracking*" [Title/abstract] | 52 249 |
| 3 | 1 OR 2 | 87 769 |
|  | Infant |  |
| 4 | Infant [MeSH] OR Infant Behavior [MeSH] | 1 253 900 |
| 5 | baby* OR babies OR infancy OR infant* OR month-old* OR months-old* OR "months of age" OR newborn* OR "new born" OR "new borns" OR "newly born" OR neonat*  [Title/abstract] | 979 623 |
| 6 | 4 OR 5 | 1 667 770 |
| 7 | 3 AND 6 | 5 465 |

**Table SM3.** *Literature search in Scopus 2023-08-21.*

| Search number | Search term | Number references |
| --- | --- | --- |
|  | Eye-tracking |  |
| 1 | TITLE-ABS-KEY ( "eye follow*" )  OR  TITLE-ABS-KEY ( eyemovement* )  OR  TITLE-ABS-KEY ( eye-movement* )  OR  TITLE-ABS-KEY ( eyetracker )  OR  TITLE-ABS-KEY ( "eye tracker" )  OR  TITLE-ABS-KEY ( eyetracking* )  OR  TITLE-ABS-KEY ( eye-tracking* )  OR  TITLE-ABS-KEY ( "gaze follow*" )  OR  TITLE-ABS-KEY ( "gaze instability" )  OR  TITLE-ABS-KEY ( "gaze stability" )  OR  TITLE-ABS-KEY ( "gaze stabilization" )  OR  TITLE-ABS-KEY ( "gaze tracked" )  OR  TITLE-ABS-KEY ( gazetracking )  OR  TITLE-ABS-KEY ( "gaze tracking" )  OR  TITLE-ABS-KEY ( "infant gaze" )  OR  TITLE-ABS-KEY ( "infants gaze" )  OR  TITLE-ABS-KEY ( "ocular mot*" )  OR  TITLE-ABS-KEY ( "oculomotor function*" )  OR  TITLE-ABS-KEY ( "oculomotor dysfunction*" )  OR  TITLE-ABS-KEY ( "oculomotor track*" )  OR  TITLE-ABS-KEY ( "visual tracking*" ) | 136 641 |
|  | Infant |  |
| 2 | TITLE-ABS-KEY ( baby* )  OR  TITLE-ABS-KEY ( babies )  OR  TITLE-ABS-KEY ( infancy )  OR  TITLE-ABS-KEY ( infant* )  OR  TITLE-ABS-KEY ( month-old* )  OR  TITLE-ABS-KEY ( months-old* )  OR  TITLE-ABS-KEY ( "months of age" )  OR  TITLE-ABS-KEY ( newborn* )  OR  TITLE-ABS-KEY ( "new born" )  OR  TITLE-ABS-KEY ( "new borns" )  OR  TITLE-ABS-KEY ( "newly born" )  OR  TITLE-ABS-KEY ( neonat* ) | 2 102 124 |
| 3 | 1 AND 2 | 6 914 |

**Table SM4.** *List of all keywords from articles’ metadata.*

| Rank | Nr occurences | Keyword |
| --- | --- | --- |
| #1 | 1076 | physiology |
| #2 | 762 | infant |
| #3 | 556 | humans |
| #4 | 454 | female |
| #5 | 439 | male |
| #6 | 277 | attention |
| #7 | 244 | eye movements |
| #8 | 227 | child |
| #9 | 170 | psychology |
| #10 | 164 | child development |
| #11 | 158 | ocular |
| #12 | 148 | visual perception |
| #13 | 143 | fixation |
| #14 | 125 | eye tracking |
| #15 | 122 | photic stimulation |
| #16 | 116 | preschool |
| #17 | 107 | cues |
| #18 | 102 | eye-tracking |
| #19 | 101 | learning |
| #20 | 97 | adult |
| #21 | 95 | infancy |
| #22 | 89 | infant development |
| #23 | 89 | language development |
| #24 | 77 | pattern recognition |
| #25 | 73 | autistic disorder |
| #26 | 71 | visual |
| #27 | 69 | cognition |
| #28 | 68 | infant behavior |
| #29 | 67 | methods |
| #30 | 64 | infants |
| #31 | 62 | face |
| #32 | 61 | memory |
| #33 | 53 | physiopathology |
| #34 | 52 | psychological |
| #35 | 51 | eye movement measurements |
| #36 | 51 | saccades |
| #37 | 50 | autism spectrum disorder |
| #38 | 50 | visual attention |
| #39 | 47 | age factors |
| #40 | 47 | language |
| #41 | 46 | newborn |
| #42 | 45 | facial expression |
| #43 | 45 | motion perception |
| #44 | 45 | orientation |
| #45 | 45 | reaction time |
| #46 | 44 | emotions |
| #47 | 44 | human |
| #48 | 44 | speech perception |
| #49 | 43 | eye fixation |
| #50 | 42 | diagnosis |
| #51 | 41 | speech |
| #52 | 39 | longitudinal studies |
| #53 | 39 | vocabulary |
| #54 | 38 | psychomotor performance |
| #55 | 37 | interpersonal relations |
| #56 | 36 | article |
| #57 | 36 | child development disorders |
| #58 | 36 | comprehension |
| #59 | 36 | pervasive |
| #60 | 35 | humanism |
| #61 | 35 | humanities |
| #62 | 33 | communication |
| #63 | 33 | social behavior |
| #64 | 32 | anticipation |
| #65 | 32 | social perception |
| #66 | 32 | young adult |
| #67 | 31 | facial recognition |
| #68 | 31 | pupil |
| #69 | 30 | face perception |
| #70 | 30 | visual tracking |
| #71 | 29 | fear |
| #72 | 29 | recognition |
| #73 | 29 | vision |
| #74 | 28 | concept formation |
| #75 | 28 | premature |
| #76 | 28 | pursuit |
| #77 | 28 | smooth |
| #78 | 28 | time factors |
| #79 | 26 | eye-tracking technology |
| #80 | 26 | social cognition |
| #81 | 25 | only child |
| #82 | 24 | autism |
| #83 | 24 | genetics |
| #84 | 23 | bias (epidemiology) |
| #85 | 22 | brain |
| #86 | 22 | cognitive development |
| #87 | 22 | verbal learning |
| #88 | 21 | aging |
| #89 | 21 | analysis of variance |
| #90 | 21 | development |
| #91 | 20 | movement |
| #92 | 20 | semantics |
| #93 | 19 | animals |
| #94 | 19 | eye movement |
| #95 | 19 | face processing |
| #96 | 19 | goals |
| #97 | 19 | human experiment |
| #98 | 19 | parents |
| #99 | 19 | temperament |
| #100 | 19 | word learning |
| #101 | 18 | controlled study |
| #102 | 18 | emotion |
| #103 | 18 | epidemiology |
| #104 | 18 | evoked potentials |
| #105 | 18 | happiness |
| #106 | 18 | head movements |
| #107 | 18 | language acquisition |
| #108 | 18 | short-term |
| #109 | 17 | attentional bias |
| #110 | 16 | continental population groups |
| #111 | 16 | gaze following |
| #112 | 16 | mothers |
| #113 | 15 | arousal |
| #114 | 15 | discrimination |
| #115 | 15 | eye |
| #116 | 15 | perception |
| #117 | 15 | phonetics |
| #118 | 15 | prospective studies |
| #119 | 15 | siblings |
| #120 | 14 | acoustic stimulation |
| #121 | 14 | gestures |
| #122 | 14 | mother-child relations |
| #123 | 14 | pathology |
| #124 | 14 | preschool child |
| #125 | 14 | risk factors |
| #126 | 14 | space perception |
| #127 | 14 | visual acuity |
| #128 | 13 | anxiety |
| #129 | 13 | child behavior |
| #130 | 13 | electrooculography |
| #131 | 13 | intention |
| #132 | 13 | mouth |
| #133 | 13 | play and playthings |
| #134 | 13 | visual fields |
| #135 | 12 | anger |
| #136 | 12 | auditory perception |
| #137 | 12 | executive function |
| #138 | 12 | growth & development |
| #139 | 12 | individuality |
| #140 | 12 | models |
| #141 | 12 | multilingualism |
| #142 | 12 | neuropsychological tests |
| #143 | 12 | pupillometry |
| #144 | 12 | reproducibility of results |
| #145 | 12 | sex characteristics |
| #146 | 11 | adolescent |
| #147 | 11 | eye gaze |
| #148 | 11 | gestational age |
| #149 | 11 | imitative behavior |
| #150 | 11 | locomotion |
| #151 | 11 | motor activity |
| #152 | 11 | selective attention |
| #153 | 10 | association learning |
| #154 | 10 | behavior |
| #155 | 10 | cross-sectional studies |
| #156 | 10 | electroencephalography |
| #157 | 10 | gaze |
| #158 | 10 | genetic predisposition to disease |
| #159 | 10 | habituation |
| #160 | 10 | joint attention |
| #161 | 10 | linguistics |
| #162 | 10 | motivation |
| #163 | 10 | prediction |
| #164 | 10 | robotics |
| #165 | 9 | affect |
| #166 | 9 | age differences |
| #167 | 9 | autism spectrum disorders |
| #168 | 9 | caregivers |
| #169 | 9 | choice behavior |
| #170 | 9 | color |
| #171 | 9 | discrimination (psychology) |
| #172 | 9 | discrimination learning |
| #173 | 9 | form perception |
| #174 | 9 | pregnancy |
| #175 | 9 | social attention |
| #176 | 9 | social interaction |
| #177 | 9 | visual stimulation |
| #178 | 8 | action prediction |
| #179 | 8 | bilingualism |
| #180 | 8 | case-control studies |
| #181 | 8 | child language |
| #182 | 8 | developmental disabilities |
| #183 | 8 | facial expressions |
| #184 | 8 | hand |
| #185 | 8 | longitudinal study |
| #186 | 8 | motor skills |
| #187 | 8 | normal human |
| #188 | 8 | oral communication |
| #189 | 8 | phenotype |
| #190 | 8 | priority journal |
| #191 | 8 | statistical |
| #192 | 8 | task performance |
| #193 | 8 | word recognition |
| #194 | 7 | action understanding |
| #195 | 7 | clinical article |
| #196 | 7 | depth perception |
| #197 | 7 | follow-up studies |
| #198 | 7 | fragile x syndrome |
| #199 | 7 | head |
| #200 | 7 | major clinical study |
| #201 | 7 | middle aged |
| #202 | 7 | nystagmus |
| #203 | 7 | parent-child relations |
| #204 | 7 | posture |
| #205 | 7 | psychophysiologic |
| #206 | 7 | sleep |
| #207 | 7 | social development |
| #208 | 7 | social environment |
| #209 | 7 | statistical learning |
| #210 | 7 | surveys and questionnaires |
| #211 | 7 | toddlers |
| #212 | 7 | video recording |
| #213 | 7 | vision tests |
| #214 | 7 | white people |
| #215 | 6 | aged |
| #216 | 6 | asian people |
| #217 | 6 | binocular |
| #218 | 6 | dogs |
| #219 | 6 | eeg |
| #220 | 6 | exploratory behavior |
| #221 | 6 | face scanning |
| #222 | 6 | hand strength |
| #223 | 6 | individual differences |
| #224 | 6 | inhibition |
| #225 | 6 | language development disorders |
| #226 | 6 | preferences |
| #227 | 6 | problem solving |
| #228 | 6 | psychological tests |
| #229 | 6 | sex factors |
| #230 | 6 | sign language |
| #231 | 6 | social skills |
| #232 | 6 | sound |
| #233 | 6 | theory of mind |
| #234 | 6 | voice |
| #235 | 6 | walking |
| #236 | 5 | asd |
| #237 | 5 | association |
| #238 | 5 | auditory stimulation |
| #239 | 5 | categorization |
| #240 | 5 | china |
| #241 | 5 | cohort studies |
| #242 | 5 | color perception |
| #243 | 5 | contrast sensitivity |
| #244 | 5 | culture |
| #245 | 5 | diagnostic imaging |
| #246 | 5 | ethnology |
| #247 | 5 | executive attention |
| #248 | 5 | eyetracking |
| #249 | 5 | high-risk infants |
| #250 | 5 | infant cognition |
| #251 | 5 | infant memory |
| #252 | 5 | low birth weight |
| #253 | 5 | malawi |
| #254 | 5 | mathematics |
| #255 | 5 | mental recall |
| #256 | 5 | motor development |
| #257 | 5 | orienting |
| #258 | 5 | other-race effect |
| #259 | 5 | oxytocin |
| #260 | 5 | perceptual masking |
| #261 | 5 | photostimulation |
| #262 | 5 | prematurity |
| #263 | 5 | psychophysics |
| #264 | 5 | racial groups |
| #265 | 5 | random allocation |
| #266 | 5 | sex differences |
| #267 | 5 | spatial |
| #268 | 5 | statistics & numerical data |
| #269 | 5 | stimulus |
| #270 | 5 | toddler |
| #271 | 5 | very low birth weight |
| #272 | 5 | visual development |
| #273 | 5 | visual search |
| #274 | 5 | williams syndrome |
| #275 | 5 | words (phonetic units) |
| #276 | 4 | adults |
| #277 | 4 | age |
| #278 | 4 | alerting |
| #279 | 4 | animal |
| #280 | 4 | associative learning |
| #281 | 4 | attention deficit disorder with hyperactivity |
| #282 | 4 | audiovisual speech |
| #283 | 4 | audiovisual speech perception |
| #284 | 4 | brain mapping |
| #285 | 4 | cats |
| #286 | 4 | cerebral cortex |
| #287 | 4 | chemically induced |
| #288 | 4 | children |
| #289 | 4 | data interpretation |
| #290 | 4 | down syndrome |
| #291 | 4 | early development |
| #292 | 4 | emotion processing |
| #293 | 4 | extremely premature |
| #294 | 4 | eye contact |
| #295 | 4 | fear bias |
| #296 | 4 | fnirs |
| #297 | 4 | form and shape perception |
| #298 | 4 | generalization |
| #299 | 4 | h-reflex |
| #300 | 4 | heart rate |
| #301 | 4 | infant attention |
| #302 | 4 | infant siblings |
| #303 | 4 | instrumentation |
| #304 | 4 | judgment |
| #305 | 4 | linear models |
| #306 | 4 | longitudinal |
| #307 | 4 | maternal behavior |
| #308 | 4 | mental processes |
| #309 | 4 | motion |
| #310 | 4 | motion pictures |
| #311 | 4 | neurodevelopment |
| #312 | 4 | no terms assigned |
| #313 | 4 | object attachment |
| #314 | 4 | object recognition |
| #315 | 4 | observation |
| #316 | 4 | predictive value of tests |
| #317 | 4 | premature birth |
| #318 | 4 | pupil dilation |
| #319 | 4 | race |
| #320 | 4 | reference values |
| #321 | 4 | reflex |
| #322 | 4 | reliability |
| #323 | 4 | risk assessment |
| #324 | 4 | rotation |
| #325 | 4 | saccadic eye movement |
| #326 | 4 | scene perception |
| #327 | 4 | sibling |
| #328 | 4 | social learning |
| #329 | 4 | social orienting |
| #330 | 4 | statistics as topic |
| #331 | 4 | stimulus response |
| #332 | 4 | visual memory |
| #333 | 4 | visual orientation |
| #334 | 4 | visual scanning |
| #335 | 4 | wakefulness |
| #336 | 3 | action |
| #337 | 3 | action perception |
| #338 | 3 | alertness |
| #339 | 3 | animal shells |
| #340 | 3 | anxiety disorders |
| #341 | 3 | aptitude |
| #342 | 3 | attention bias |
| #343 | 3 | attention capture |
| #344 | 3 | autism spectrum disorder (asd) |
| #345 | 3 | biological motion |
| #346 | 3 | biomechanical phenomena |
| #347 | 3 | brain development |
| #348 | 3 | childhood development |
| #349 | 3 | cognitive control |
| #350 | 3 | computer-assisted |
| #351 | 3 | conceptual development |
| #352 | 3 | cross-cultural comparison |
| #353 | 3 | depression |
| #354 | 3 | early diagnosis |
| #355 | 3 | ethnicity |
| #356 | 3 | expectations |
| #357 | 3 | eye‐tracking |
| #358 | 3 | face preference |
| #359 | 3 | faces |
| #360 | 3 | fast mapping |
| #361 | 3 | feasibility studies |
| #362 | 3 | finland |
| #363 | 3 | forecasting |
| #364 | 3 | functional laterality |
| #365 | 3 | gaze direction |
| #366 | 3 | gender |
| #367 | 3 | head movement |
| #368 | 3 | heart block |
| #369 | 3 | human information storage |
| #370 | 3 | infant eye movements |
| #371 | 3 | infant learning |
| #372 | 3 | infant perception |
| #373 | 3 | information processing |
| #374 | 3 | interaction |
| #375 | 3 | japan |
| #376 | 3 | labeling |
| #377 | 3 | lexical development |
| #378 | 3 | maternal sensitivity |
| #379 | 3 | metabolism |
| #380 | 3 | movement (physiology) |
| #381 | 3 | mutual exclusivity |
| #382 | 3 | neurodevelopmental disorders |
| #383 | 3 | nonverbal communication |
| #384 | 3 | object tracking |
| #385 | 3 | occlusion |
| #386 | 3 | optokinetic |
| #387 | 3 | ostensive cues |
| #388 | 3 | perceptual development |
| #389 | 3 | perceptual narrowing |
| #390 | 3 | photography |
| #391 | 3 | play |
| #392 | 3 | pointing |
| #393 | 3 | preferential looking |
| #394 | 3 | prefrontal cortex |
| #395 | 3 | pregnancy complications |
| #396 | 3 | probability |
| #397 | 3 | prosocial behavior |
| #398 | 3 | prospective study |
| #399 | 3 | regression analysis |
| #400 | 3 | retina |
| #401 | 3 | reward |
| #402 | 3 | risk |
| #403 | 3 | saliency |
| #404 | 3 | self-control |
| #405 | 3 | sensitivity and specificity |
| #406 | 3 | size perception |
| #407 | 3 | social interactions |
| #408 | 3 | socioeconomic factors |
| #409 | 3 | spatial behavior |
| #410 | 3 | task performance and analysis |
| #411 | 3 | teaching |
| #412 | 3 | test construction |
| #413 | 3 | third-party interactions |
| #414 | 3 | turn taking |
| #415 | 3 | verb learning |
| #416 | 3 | verbs |
| #417 | 3 | vision disorders |
| #418 | 3 | visual cortex |
| #419 | 3 | visual discrimination |
| #420 | 3 | visual displays |
| #421 | 3 | visual preference |
| #422 | 3 | visual short-term memory |
| #423 | 3 | visual working memory |
| #424 | 3 | vocalization |
| #425 | 3 | vowels |
| #426 | 3 | word comprehension |
| #427 | 3 | working memory |
| #428 | 2 | abstraction |
| #429 | 2 | action observation |
| #430 | 2 | adaptation |
| #431 | 2 | adult attitudes |
| #432 | 2 | adult development |
| #433 | 2 | adverse effects |
| #434 | 2 | age distribution |
| #435 | 2 | altruism |
| #436 | 2 | anatomy & histology |
| #437 | 2 | anticipatory looking |
| #438 | 2 | anticonvulsants |
| #439 | 2 | approximate number system |
| #440 | 2 | asian continental ancestry group |
| #441 | 2 | at risk populations |
| #442 | 2 | attachment |
| #443 | 2 | attachment behavior |
| #444 | 2 | attention deficit disorder |
| #445 | 2 | attention to threat |
| #446 | 2 | audio-visual matching |
| #447 | 2 | audiovisual speech integration |
| #448 | 2 | awareness |
| #449 | 2 | bayes theorem |
| #450 | 2 | bhutan |
| #451 | 2 | bias |
| #452 | 2 | binocular vision |
| #453 | 2 | biomarker |
| #454 | 2 | birth weight |
| #455 | 2 | black people |
| #456 | 2 | blinking |
| #457 | 2 | calibration |
| #458 | 2 | cartoons |
| #459 | 2 | category learning |
| #460 | 2 | causality |
| #461 | 2 | checklist |
| #462 | 2 | chi-square distribution |
| #463 | 2 | cognitive processes |
| #464 | 2 | conditioning |
| #465 | 2 | consonants |
| #466 | 2 | convergence |
| #467 | 2 | conversation |
| #468 | 2 | cooperative behavior |
| #469 | 2 | covid-19 |
| #470 | 2 | crying |
| #471 | 2 | data analysis |
| #472 | 2 | deafness |
| #473 | 2 | deferred imitation |
| #474 | 2 | developmental changes |
| #475 | 2 | dissociative disorders |
| #476 | 2 | dna methylation |
| #477 | 2 | drug effects |
| #478 | 2 | drug therapy |
| #479 | 2 | educational status |
| #480 | 2 | effortful control |
| #481 | 2 | eggs |
| #482 | 2 | electrodiagnosis |
| #483 | 2 | emotional development |
| #484 | 2 | encoding |
| #485 | 2 | endogenous attention |
| #486 | 2 | endophenotype |
| #487 | 2 | endophenotypes |
| #488 | 2 | environment |
| #489 | 2 | epilepsy |
| #490 | 2 | episodic memory |
| #491 | 2 | european continental ancestry group |
| #492 | 2 | event related potential |
| #493 | 2 | event segmentation |
| #494 | 2 | event-related potentials |
| #495 | 2 | event-related potentials (erps) |
| #496 | 2 | experience |
| #497 | 2 | eye fixations |
| #498 | 2 | eye-tracker |
| #499 | 2 | face discrimination |
| #500 | 2 | face race |
| #501 | 2 | face-processing |
| #502 | 2 | factor analysis |
| #503 | 2 | familiarity |
| #504 | 2 | family |
| #505 | 2 | feeding behavior |
| #506 | 2 | fixation duration |
| #507 | 2 | fixation durations |
| #508 | 2 | focused attention |
| #509 | 2 | frontal lobe |
| #510 | 2 | gaze behavior |
| #511 | 2 | gaze cueing |
| #512 | 2 | gaze fixation |
| #513 | 2 | gaze-following |
| #514 | 2 | gazing |
| #515 | 2 | germany |
| #516 | 2 | gesture |
| #517 | 2 | goal anticipation |
| #518 | 2 | goal prediction speed |
| #519 | 2 | goal-based action predictions |
| #520 | 2 | grammar |
| #521 | 2 | groups by age |
| #522 | 2 | health resources |
| #523 | 2 | hearing |
| #524 | 2 | helping behavior |
| #525 | 2 | human sex differences |
| #526 | 2 | humanoid robot |
| #527 | 2 | infant eye tracking |
| #528 | 2 | infant stress perception |
| #529 | 2 | infant-directed speech |
| #530 | 2 | infants at risk for asd |
| #531 | 2 | initiating joint attention |
| #532 | 2 | intonation |
| #533 | 2 | knowledge |
| #534 | 2 | language ability |
| #535 | 2 | language comprehension |
| #536 | 2 | language processing |
| #537 | 2 | language tests |
| #538 | 2 | latent period |
| #539 | 2 | lexical access |
| #540 | 2 | lexical processing |
| #541 | 2 | long-term |
| #542 | 2 | looking behavior |
| #543 | 2 | looking time |
| #544 | 2 | macaca mulatta |
| #545 | 2 | magnetic resonance imaging |
| #546 | 2 | markov chains |
| #547 | 2 | maternal exposure |
| #548 | 2 | maternal stress |
| #549 | 2 | mexico |
| #550 | 2 | mice |
| #551 | 2 | mother child relations |
| #552 | 2 | motor performance |
| #553 | 2 | motor processes |
| #554 | 2 | motor system |
| #555 | 2 | movement perception |
| #556 | 2 | msh release-inhibiting hormone |
| #557 | 2 | multimodal |
| #558 | 2 | multimodal perception |
| #559 | 2 | multisensory perception |
| #560 | 2 | music |
| #561 | 2 | narratives |
| #562 | 2 | natural pedagogy |
| #563 | 2 | near-infrared |
| #564 | 2 | negative emotion |
| #565 | 2 | negativity bias |
| #566 | 2 | nerve net |
| #567 | 2 | neural pathways |
| #568 | 2 | neurons |
| #569 | 2 | nonhuman |
| #570 | 2 | nouns |
| #571 | 2 | object |
| #572 | 2 | object learning |
| #573 | 2 | object processing |
| #574 | 2 | objects |
| #575 | 2 | oculomotor muscles |
| #576 | 2 | optic flow |
| #577 | 2 | optical illusions |
| #578 | 2 | orienting response |
| #579 | 2 | other-race |
| #580 | 2 | outcome assessment |
| #581 | 2 | own-species bias |
| #582 | 2 | pandemics |
| #583 | 2 | parent |
| #584 | 2 | parental stress |
| #585 | 2 | perceptual closure |
| #586 | 2 | personality |
| #587 | 2 | phonology |
| #588 | 2 | physiological |
| #589 | 2 | postnatal period |
| #590 | 2 | practice |
| #591 | 2 | preference |
| #592 | 2 | prenatal exposure delayed effects |
| #593 | 2 | preterm |
| #594 | 2 | preterm infants |
| #595 | 2 | prevention & control |
| #596 | 2 | probability learning |
| #597 | 2 | processing speed |
| #598 | 2 | prospective reasoning |
| #599 | 2 | protective factors |
| #600 | 2 | psycholinguistics |
| #601 | 2 | pupil diameter |
| #602 | 2 | pupil reflex |
| #603 | 2 | questionnaire |
| #604 | 2 | reaching |
| #605 | 2 | receptive language |
| #606 | 2 | recognition (psychology) |
| #607 | 2 | reinforcement |
| #608 | 2 | relational memory |
| #609 | 2 | replication |
| #610 | 2 | retention |
| #611 | 2 | rhythm |
| #612 | 2 | risk factor |
| #613 | 2 | scene viewing |
| #614 | 2 | self-regulation |
| #615 | 2 | sequence learning |
| #616 | 2 | severity of illness index |
| #617 | 2 | sex difference |
| #618 | 2 | short term memory |
| #619 | 2 | smell |
| #620 | 2 | smiling |
| #621 | 2 | social cognitive development |
| #622 | 2 | social competence |
| #623 | 2 | social context |
| #624 | 2 | social cues |
| #625 | 2 | social stimuli |
| #626 | 2 | social-cognitive development |
| #627 | 2 | sociality |
| #628 | 2 | spatial processing |
| #629 | 2 | spectroscopy |
| #630 | 2 | speech development |
| #631 | 2 | speech perception development |
| #632 | 2 | speech processing |
| #633 | 2 | statistics |
| #634 | 2 | support |
| #635 | 2 | surgency |
| #636 | 2 | sweden |
| #637 | 2 | symptom |
| #638 | 2 | synchrony |
| #639 | 2 | talking face |
| #640 | 2 | temporal contingency |
| #641 | 2 | thalamus |
| #642 | 2 | theoretical |
| #643 | 2 | thinking |
| #644 | 2 | threat |
| #645 | 2 | tool use |
| #646 | 2 | tool use behavior |
| #647 | 2 | toxicity |
| #648 | 2 | tracking |
| #649 | 2 | trends |
| #650 | 2 | turn-taking |
| #651 | 2 | uncertainty |
| #652 | 2 | updating |
| #653 | 2 | verbalization |
| #654 | 2 | vestibulo-ocular |
| #655 | 2 | visual expectations |
| #656 | 2 | visual exploration |
| #657 | 2 | visual field |
| #658 | 2 | visual orienting |
| #659 | 2 | visual pathways |
| #660 | 2 | visual system |
| #661 | 2 | visually impaired persons |
| #662 | 2 | word order |
| #663 | 1 | 1 vs 2 vs 4 mo old infants |
| #664 | 1 | 11–17 wk olds |
| #665 | 1 | 13- to 15-month-old infants |
| #666 | 1 | 14-month-old infants |
| #667 | 1 | 18-month-olds |
| #668 | 1 | 19 day to 1 yr olds vs adults |
| #669 | 1 | 1–3 day olds |
| #670 | 1 | 1–3 mo olds |
| #671 | 1 | 3d coherence |
| #672 | 1 | 3d form recognition |
| #673 | 1 | a-not-b task |
| #674 | 1 | ability to fixate & track |
| #675 | 1 | absent entities |
| #676 | 1 | absent objects |
| #677 | 1 | absent reference |
| #678 | 1 | abstract grammatical knowledge |
| #679 | 1 | abstract knowledge |
| #680 | 1 | accommodation |
| #681 | 1 | accumulation |
| #682 | 1 | acoustic packages |
| #683 | 1 | acoustic packaging |
| #684 | 1 | actigraphy |
| #685 | 1 | action anticipation |
| #686 | 1 | action control |
| #687 | 1 | action effect bindings |
| #688 | 1 | action interpretation |
| #689 | 1 | action mirroring |
| #690 | 1 | action priming |
| #691 | 1 | action semantics |
| #692 | 1 | action sequences |
| #693 | 1 | actions |
| #694 | 1 | active experience |
| #695 | 1 | active movement |
| #696 | 1 | activity level |
| #697 | 1 | addition |
| #698 | 1 | adhd |
| #699 | 1 | adult model's eye gaze |
| #700 | 1 | adult shifts |
| #701 | 1 | aed |
| #702 | 1 | affiliation inference |
| #703 | 1 | affordance learning |
| #704 | 1 | african american |
| #705 | 1 | age groups |
| #706 | 1 | age of onset |
| #707 | 1 | agency |
| #708 | 1 | alexnet |
| #709 | 1 | algorithms |
| #710 | 1 | alleles |
| #711 | 1 | alzheimer disease |
| #712 | 1 | ambient and focal processing |
| #713 | 1 | amygdala |
| #714 | 1 | android |
| #715 | 1 | animal experiment |
| #716 | 1 | ans |
| #717 | 1 | anthropometry |
| #718 | 1 | anticipatory eye movements |
| #719 | 1 | anticipatory gaze |
| #720 | 1 | anticipatory gaze shifts |
| #721 | 1 | anticipatory looking paradigm |
| #722 | 1 | antisocial personality disorder |
| #723 | 1 | apolipoprotein a-i |
| #724 | 1 | apolipoprotein c-i |
| #725 | 1 | apolipoprotein e4 |
| #726 | 1 | apolipoproteins |
| #727 | 1 | apolipoproteins a |
| #728 | 1 | apolipoproteins d |
| #729 | 1 | apoprotein(a) |
| #730 | 1 | appetitive behavior |
| #731 | 1 | arbitrary versus functional actions |
| #732 | 1 | articulation (speech) |
| #733 | 1 | associative processes |
| #734 | 1 | at-risk |
| #735 | 1 | attachment security |
| #736 | 1 | attachment style |
| #737 | 1 | attachment styles |
| #738 | 1 | attention bias to threat |
| #739 | 1 | attention control training |
| #740 | 1 | attention development |
| #741 | 1 | attention direction |
| #742 | 1 | attention disengagement |
| #743 | 1 | attention holding |
| #744 | 1 | attention network test |
| #745 | 1 | attention switching |
| #746 | 1 | attention test |
| #747 | 1 | attention to eyes |
| #748 | 1 | attentional capture |
| #749 | 1 | attentional control |
| #750 | 1 | attentional cueing |
| #751 | 1 | attentional cues |
| #752 | 1 | attentional processes |
| #753 | 1 | attentional synchrony |
| #754 | 1 | attitude to health |
| #755 | 1 | attractiveness |
| #756 | 1 | attunement |
| #757 | 1 | atypical development |
| #758 | 1 | atypicality |
| #759 | 1 | audiences |
| #760 | 1 | audio-visual redundancy |
| #761 | 1 | audio-visual speech perception |
| #762 | 1 | audiovisual integration |
| #763 | 1 | audiovisual mismatch response |
| #764 | 1 | audiovisual perception |
| #765 | 1 | audiovisual processing |
| #766 | 1 | audiovisual speech comprehension |
| #767 | 1 | auditory acuity |
| #768 | 1 | auditory discrimination |
| #769 | 1 | auditory information |
| #770 | 1 | auditory speech |
| #771 | 1 | auditory threshold |
| #772 | 1 | auditory visual matching |
| #773 | 1 | auditory-visual asynchrony |
| #774 | 1 | auditory-visual speech benefit |
| #775 | 1 | auditory-visual speech perception |
| #776 | 1 | automation |
| #777 | 1 | autonomic arousal |
| #778 | 1 | autonomic nervous system |
| #779 | 1 | autonomic nervous system function |
| #780 | 1 | avoidance learning |
| #781 | 1 | baby media |
| #782 | 1 | baby sibs |
| #783 | 1 | baby signs |
| #784 | 1 | backward inhibition |
| #785 | 1 | bayes factor analysis |
| #786 | 1 | beauty |
| #787 | 1 | behavior control |
| #788 | 1 | behavior observation techniques |
| #789 | 1 | behavioral genetics |
| #790 | 1 | behavioral reinforcement |
| #791 | 1 | behavioral research |
| #792 | 1 | beliefs |
| #793 | 1 | biased attention |
| #794 | 1 | bilingual advantage |
| #795 | 1 | bimodal bilingualism |
| #796 | 1 | binding |
| #797 | 1 | biological |
| #798 | 1 | biological markers |
| #799 | 1 | biomarkers |
| #800 | 1 | biomechanics |
| #801 | 1 | blindness |
| #802 | 1 | body |
| #803 | 1 | body actions |
| #804 | 1 | body movement |
| #805 | 1 | body orientation |
| #806 | 1 | body representations |
| #807 | 1 | body shape |
| #808 | 1 | brain depth stimulation |
| #809 | 1 | brain hemorrhage |
| #810 | 1 | broader autism phenotype |
| #811 | 1 | buserelin |
| #812 | 1 | california |
| #813 | 1 | cantonese |
| #814 | 1 | capsules |
| #815 | 1 | case control study |
| #816 | 1 | catechol o-methyltransferase |
| #817 | 1 | categorical perception |
| #818 | 1 | categorisation |
| #819 | 1 | caucasian |
| #820 | 1 | center bias |
| #821 | 1 | central nervous system |
| #822 | 1 | change detection |
| #823 | 1 | change-detection |
| #824 | 1 | chasing |
| #825 | 1 | child behavior checklist |
| #826 | 1 | child behavior disorders |
| #827 | 1 | child care |
| #828 | 1 | child parent relation |
| #829 | 1 | child rearing |
| #830 | 1 | children born preterm |
| #831 | 1 | children's development |
| #832 | 1 | chorioamnionitis |
| #833 | 1 | chrna4 |
| #834 | 1 | circadian rhythm |
| #835 | 1 | classical |
| #836 | 1 | classification (cognitive process) |
| #837 | 1 | clinical assessment |
| #838 | 1 | clinical outcome |
| #839 | 1 | clinical trial |
| #840 | 1 | coarticulation |
| #841 | 1 | code switching |
| #842 | 1 | cognition disorders |
| #843 | 1 | cognitive bias |
| #844 | 1 | cognitive dysfunction |
| #845 | 1 | cognitive effort |
| #846 | 1 | cognitive function test |
| #847 | 1 | cognitive generalization |
| #848 | 1 | cognitive neuroscience |
| #849 | 1 | cognitive systems |
| #850 | 1 | cohesion |
| #851 | 1 | cohort analysis |
| #852 | 1 | collaboration |
| #853 | 1 | color vision |
| #854 | 1 | color words |
| #855 | 1 | colour labels |
| #856 | 1 | coloured liquid laundry detergent capsules |
| #857 | 1 | communication disorder |
| #858 | 1 | communicative cues |
| #859 | 1 | communicative demonstration |
| #860 | 1 | communicative development |
| #861 | 1 | comparative psychology |
| #862 | 1 | comparative study |
| #863 | 1 | comparison |
| #864 | 1 | comparison study |
| #865 | 1 | complementary feeding |
| #866 | 1 | complex visual array |
| #867 | 1 | complications |
| #868 | 1 | comprehensive comparisons |
| #869 | 1 | computational modeling |
| #870 | 1 | computer |
| #871 | 1 | computerised cognitive training |
| #872 | 1 | computers |
| #873 | 1 | comt |
| #874 | 1 | conditions of occlusion |
| #875 | 1 | conflict |
| #876 | 1 | conflicting social cues |
| #877 | 1 | consciousness |
| #878 | 1 | consistent performance |
| #879 | 1 | consonant bias |
| #880 | 1 | conspec |
| #881 | 1 | contingency management |
| #882 | 1 | continuous circular motion |
| #883 | 1 | controlled clinical trial |
| #884 | 1 | conversations |
| #885 | 1 | convolutional neural networks (cnns) |
| #886 | 1 | cooperation |
| #887 | 1 | coordinated behavior |
| #888 | 1 | coordination |
| #889 | 1 | corneal-reflection technique |
| #890 | 1 | corpus callosum |
| #891 | 1 | correlational study |
| #892 | 1 | cortical tracking |
| #893 | 1 | crawling |
| #894 | 1 | cross cultural differences |
| #895 | 1 | cross-correlation |
| #896 | 1 | cross-cultural study |
| #897 | 1 | cross-linguistic study |
| #898 | 1 | cross-modal matching |
| #899 | 1 | cross-sectional study |
| #900 | 1 | crowding |
| #901 | 1 | cry |
| #902 | 1 | cue integration |
| #903 | 1 | cultural differences |
| #904 | 1 | danish |
| #905 | 1 | data processing |
| #906 | 1 | data quality |
| #907 | 1 | daytime sleep |
| #908 | 1 | deaf |
| #909 | 1 | deception |
| #910 | 1 | decision making |
| #911 | 1 | decreased attention |
| #912 | 1 | deep neural networks (dnns) |
| #913 | 1 | delayed match retrieval |
| #914 | 1 | denmark |
| #915 | 1 | detergents |
| #916 | 1 | developmental neurology & neurodisability |
| #917 | 1 | developmental neuroscience |
| #918 | 1 | developmental robotics |
| #919 | 1 | developmental social neuroscience |
| #920 | 1 | developmental systems |
| #921 | 1 | developmental trajectory |
| #922 | 1 | deviant-detection |
| #923 | 1 | dialect |
| #924 | 1 | diet |
| #925 | 1 | differential liability |
| #926 | 1 | diffusion magnetic resonance imaging |
| #927 | 1 | diffusion tensor imaging |
| #928 | 1 | digital video |
| #929 | 1 | dilatation |
| #930 | 1 | direct matching |
| #931 | 1 | disabled persons |
| #932 | 1 | disambiguation |
| #933 | 1 | disease classification |
| #934 | 1 | disease progression |
| #935 | 1 | disease severity |
| #936 | 1 | disease susceptibility |
| #937 | 1 | diseases |
| #938 | 1 | disengagement |
| #939 | 1 | distracter referent |
| #940 | 1 | distributional information |
| #941 | 1 | distributional learning |
| #942 | 1 | dna (cytosine-5-)-methyltransferase |
| #943 | 1 | dog |
| #944 | 1 | dorsal-ventral |
| #945 | 1 | dot-probe |
| #946 | 1 | drug use |
| #947 | 1 | dwell time |
| #948 | 1 | dyadic coordination |
| #949 | 1 | dynamic |
| #950 | 1 | dynamic couplings |
| #951 | 1 | dynamic events |
| #952 | 1 | dynamic expressions |
| #953 | 1 | dynamic faces |
| #954 | 1 | dynamic gestures |
| #955 | 1 | dynamic material |
| #956 | 1 | dynamic messages |
| #957 | 1 | dynamic object processing |
| #958 | 1 | dynamic visual cues |
| #959 | 1 | dynamic visual search |
| #960 | 1 | early childhood |
| #961 | 1 | early communication |
| #962 | 1 | early detection |
| #963 | 1 | early identification |
| #964 | 1 | early language acquisition |
| #965 | 1 | early language processing |
| #966 | 1 | early lexical comprehension |
| #967 | 1 | early lexicon |
| #968 | 1 | early literacy |
| #969 | 1 | early sociomoral evaluation |
| #970 | 1 | early word learning |
| #971 | 1 | ecologically valid method |
| #972 | 1 | educational media |
| #973 | 1 | efferent pathways |
| #974 | 1 | efficiency |
| #975 | 1 | electric impedance |
| #976 | 1 | embodied cognition |
| #977 | 1 | embodiment |
| #978 | 1 | emi |
| #979 | 1 | emotion expression |
| #980 | 1 | emotion expression processing |
| #981 | 1 | emotion expressions |
| #982 | 1 | emotion matching |
| #983 | 1 | emotion reciprocity |
| #984 | 1 | emotion recognition |
| #985 | 1 | emotion understanding |
| #986 | 1 | emotional content |
| #987 | 1 | emotional expression |
| #988 | 1 | emotional expressions |
| #989 | 1 | emotional intelligence |
| #990 | 1 | emotional mimicry |
| #991 | 1 | emotional states |
| #992 | 1 | emotionality |
| #993 | 1 | endpoints |
| #994 | 1 | entrainment |
| #995 | 1 | environmental effects |
| #996 | 1 | environmental factor |
| #997 | 1 | environmental pollutants |
| #998 | 1 | epigenesis |
| #999 | 1 | epiglottis |
| #1000 | 1 | episode |
| #1001 | 1 | episodic |
| #1002 | 1 | equipment design |
| #1003 | 1 | erp |
| #1004 | 1 | erps |
| #1005 | 1 | estonia |
| #1006 | 1 | estradiol |
| #1007 | 1 | etymology |
| #1008 | 1 | european portuguese |
| #1009 | 1 | evaluation |
| #1010 | 1 | evaluation metrics |
| #1011 | 1 | evaluation of toddler interaction |
| #1012 | 1 | evaluative conditioning |
| #1013 | 1 | event boundary |
| #1014 | 1 | event perception |
| #1015 | 1 | event related potentials |
| #1016 | 1 | event representation |
| #1017 | 1 | event-related potentials (erp) |
| #1018 | 1 | eventrepresentation |
| #1019 | 1 | executive functioning |
| #1020 | 1 | expectation |
| #1021 | 1 | experimental study |
| #1022 | 1 | experimenters |
| #1023 | 1 | experiments |
| #1024 | 1 | expressive language |
| #1025 | 1 | extraocular muscle |
| #1026 | 1 | extrapolation |
| #1027 | 1 | eye (anatomy) |
| #1028 | 1 | eye dominance |
| #1029 | 1 | eye hand coordination |
| #1030 | 1 | eye movement development |
| #1031 | 1 | eye movements during visual inspection |
| #1032 | 1 | eye status |
| #1033 | 1 | eye tracker |
| #1034 | 1 | eye-blink rate |
| #1035 | 1 | eye-hand coordination |
| #1036 | 1 | eye-mouth index |
| #1037 | 1 | eye-tracking methodology |
| #1038 | 1 | eye-tracking paradigm |
| #1039 | 1 | eyeblink reflex |
| #1040 | 1 | eyes |
| #1041 | 1 | eye‐tracking paradigm |
| #1042 | 1 | face age |
| #1043 | 1 | face and body knowledge in infancy |
| #1044 | 1 | face bias |
| #1045 | 1 | face disorder |
| #1046 | 1 | face inversion |
| #1047 | 1 | face learning |
| #1048 | 1 | face orientation |
| #1049 | 1 | face profile |
| #1050 | 1 | face recognition |
| #1051 | 1 | face scanning behavior |
| #1052 | 1 | face specialization |
| #1053 | 1 | facial feature tracking |
| #1054 | 1 | facial features |
| #1055 | 1 | fagan |
| #1056 | 1 | fairness |
| #1057 | 1 | false beliefs |
| #1058 | 1 | familial risk |
| #1059 | 1 | family expressiveness |
| #1060 | 1 | family health |
| #1061 | 1 | family history |
| #1062 | 1 | fear preference |
| #1063 | 1 | fear processing |
| #1064 | 1 | fearfulness |
| #1065 | 1 | feasibility study |
| #1066 | 1 | feature representation |
| #1067 | 1 | feature-based attention |
| #1068 | 1 | feedback |
| #1069 | 1 | field & target velocity |
| #1070 | 1 | field dependence-independence |
| #1071 | 1 | figure skating |
| #1072 | 1 | fingers |
| #1073 | 1 | first language acquisition |
| #1074 | 1 | fixation-shift-paradigm |
| #1075 | 1 | fmri |
| #1076 | 1 | follow up |
| #1077 | 1 | fourier transform |
| #1078 | 1 | france |
| #1079 | 1 | frequency |
| #1080 | 1 | frequency modulation |
| #1081 | 1 | frontal asymmetry |
| #1082 | 1 | full-term nicu graduates |
| #1083 | 1 | functional connectivity |
| #1084 | 1 | fundamental frequency |
| #1085 | 1 | g11 |
| #1086 | 1 | g14 |
| #1087 | 1 | galvanic skin response |
| #1088 | 1 | gap-overlap |
| #1089 | 1 | gaze behaviour |
| #1090 | 1 | gaze locations |
| #1091 | 1 | gaze shifts |
| #1092 | 1 | gaze stability |
| #1093 | 1 | gaze tracking |
| #1094 | 1 | gaze-contingent eye tracking |
| #1095 | 1 | gaze-contingent eye-tracking |
| #1096 | 1 | gaze-contingent eye-tracking paradigm |
| #1097 | 1 | gaze‐contingent eye‐tracking |
| #1098 | 1 | gaze‐cued attention |
| #1099 | 1 | gazing behavior |
| #1100 | 1 | gender differences |
| #1101 | 1 | gene-environment interaction |
| #1102 | 1 | genes |
| #1103 | 1 | genetic |
| #1104 | 1 | genetic variation |
| #1105 | 1 | genotype |
| #1106 | 1 | geometric form size differences |
| #1107 | 1 | geometric preference |
| #1108 | 1 | geometry |
| #1109 | 1 | gift giving |
| #1110 | 1 | give-me gesture |
| #1111 | 1 | glucocorticoids |
| #1112 | 1 | goal orientation |
| #1113 | 1 | goal type modulate |
| #1114 | 1 | goal understanding |
| #1115 | 1 | goal-directed action |
| #1116 | 1 | goal-directed gaze shifts |
| #1117 | 1 | goal-source asymmetry |
| #1118 | 1 | grammatical gender |
| #1119 | 1 | graph-based visual saliencies |
| #1120 | 1 | graphic methods |
| #1121 | 1 | habits |
| #1122 | 1 | hand (anatomy) |
| #1123 | 1 | handheld |
| #1124 | 1 | hands control |
| #1125 | 1 | hand–eye coordination |
| #1126 | 1 | haplorhini |
| #1127 | 1 | happy face |
| #1128 | 1 | head (anatomy) |
| #1129 | 1 | head orientation |
| #1130 | 1 | head-mounted camera |
| #1131 | 1 | head-mounted eye tracking |
| #1132 | 1 | head-mounted eyetracking |
| #1133 | 1 | healthy volunteers |
| #1134 | 1 | heterogeneity |
| #1135 | 1 | heterozygote |
| #1136 | 1 | high risk |
| #1137 | 1 | high risk infant |
| #1138 | 1 | high risk patient |
| #1139 | 1 | high risk-noasd |
| #1140 | 1 | hispanic |
| #1141 | 1 | hispanic or latino |
| #1142 | 1 | hiv |
| #1143 | 1 | hiv infections |
| #1144 | 1 | homeostasis |
| #1145 | 1 | horizontal bias |
| #1146 | 1 | horizontal gaze |
| #1147 | 1 | human body |
| #1148 | 1 | human faces |
| #1149 | 1 | human infants |
| #1150 | 1 | human machine systems |
| #1151 | 1 | human relation |
| #1152 | 1 | human tissue |
| #1153 | 1 | human voice |
| #1154 | 1 | hypersensitivity |
| #1155 | 1 | hypothesis |
| #1156 | 1 | hypoxic–ischemic injury |
| #1157 | 1 | iambic stress |
| #1158 | 1 | iconicity |
| #1159 | 1 | ideomotor action |
| #1160 | 1 | ideomotor theory |
| #1161 | 1 | illusions |
| #1162 | 1 | image interpretation |
| #1163 | 1 | imagination |
| #1164 | 1 | imitation (learning) |
| #1165 | 1 | imitation tasks |
| #1166 | 1 | immune response |
| #1167 | 1 | impatiens |
| #1168 | 1 | implications for fixation & attention |
| #1169 | 1 | implicit learning |
| #1170 | 1 | implicit theory of mind |
| #1171 | 1 | impossible objects |
| #1172 | 1 | impression formation |
| #1173 | 1 | individuation |
| #1174 | 1 | infant action processing |
| #1175 | 1 | infant attachment disorganization |
| #1176 | 1 | infant attention to emotional faces |
| #1177 | 1 | infant cognitive development |
| #1178 | 1 | infant communication |
| #1179 | 1 | infant event-related potentials |
| #1180 | 1 | infant eye-tracking |
| #1181 | 1 | infant face preference |
| #1182 | 1 | infant face preferences |
| #1183 | 1 | infant gaze behavior |
| #1184 | 1 | infant gaze following |
| #1185 | 1 | infant heart rate |
| #1186 | 1 | infant knowledge |
| #1187 | 1 | infant nutritional physiological phenomena |
| #1188 | 1 | infant perceptions |
| #1189 | 1 | infant pupil |
| #1190 | 1 | infant social cognition |
| #1191 | 1 | infant vision |
| #1192 | 1 | infant-directed singing |
| #1193 | 1 | infant-parent interactions dyadic interactions |
| #1194 | 1 | infants at risk |
| #1195 | 1 | infants speech perception |
| #1196 | 1 | infants’ brain responses |
| #1197 | 1 | infants’ perception |
| #1198 | 1 | infectious |
| #1199 | 1 | inference |
| #1200 | 1 | information dissemination |
| #1201 | 1 | information processing speed |
| #1202 | 1 | informed consent |
| #1203 | 1 | infrared rays |
| #1204 | 1 | inhibition (personality) |
| #1205 | 1 | inhibitory control |
| #1206 | 1 | input-specific mechanism of lexical acquisition |
| #1207 | 1 | instructional media |
| #1208 | 1 | intellectual disability |
| #1209 | 1 | intelligence |
| #1210 | 1 | intelligent agents |
| #1211 | 1 | intensive care units |
| #1212 | 1 | intention understanding |
| #1213 | 1 | intentional action |
| #1214 | 1 | inter-subject correlation |
| #1215 | 1 | interactive screen media |
| #1216 | 1 | interdependence |
| #1217 | 1 | intermodal cognition |
| #1218 | 1 | intermodal emotion matching |
| #1219 | 1 | intermodal information |
| #1220 | 1 | intermodal matching |
| #1221 | 1 | internal model |
| #1222 | 1 | internal working model |
| #1223 | 1 | internal working models |
| #1224 | 1 | interpersonal interaction |
| #1225 | 1 | interrater reliability |
| #1226 | 1 | interruption |
| #1227 | 1 | intersensory redundancy hypothesis |
| #1228 | 1 | intransitive actions |
| #1229 | 1 | irregular words |
| #1230 | 1 | isolated eyes |
| #1231 | 1 | italy |
| #1232 | 1 | japanese |
| #1233 | 1 | kabc-ii |
| #1234 | 1 | kinematics |
| #1235 | 1 | knowledge (general) |
| #1236 | 1 | knowledge representation |
| #1237 | 1 | label |
| #1238 | 1 | labels |
| #1239 | 1 | language and cognition |
| #1240 | 1 | language control |
| #1241 | 1 | language outcomes |
| #1242 | 1 | late talkers |
| #1243 | 1 | latency time |
| #1244 | 1 | lateral dominance |
| #1245 | 1 | lateralization |
| #1246 | 1 | laterally moving visual stimulus |
| #1247 | 1 | learning disabilities |
| #1248 | 1 | learning system |
| #1249 | 1 | learning systems |
| #1250 | 1 | left hemisphere |
| #1251 | 1 | leftward bias |
| #1252 | 1 | leukoencephalopathies |
| #1253 | 1 | lexical class |
| #1254 | 1 | lexical speed of processing |
| #1255 | 1 | lexicon |
| #1256 | 1 | limit of quantitation |
| #1257 | 1 | linguistic attention |
| #1258 | 1 | linguistic distance |
| #1259 | 1 | linguistic signal |
| #1260 | 1 | lipreading |
| #1261 | 1 | literacy |
| #1262 | 1 | long-term memory |
| #1263 | 1 | longevity |
| #1264 | 1 | looking behaviour |
| #1265 | 1 | looking times |
| #1266 | 1 | looking while listening |
| #1267 | 1 | looking-while-listening paradigm |
| #1268 | 1 | los angeles |
| #1269 | 1 | low birth weight infant |
| #1270 | 1 | low risk patient |
| #1271 | 1 | low risk population |
| #1272 | 1 | low-resource |
| #1273 | 1 | lung dysplasia |
| #1274 | 1 | magnitude processing |
| #1275 | 1 | mandarin chinese |
| #1276 | 1 | manual behavior |
| #1277 | 1 | manual laterality |
| #1278 | 1 | marketing |
| #1279 | 1 | markov chain |
| #1280 | 1 | markov model |
| #1281 | 1 | masks |
| #1282 | 1 | mass and size |
| #1283 | 1 | mass screening |
| #1284 | 1 | maternal anxiety |
| #1285 | 1 | maternal mental health |
| #1286 | 1 | maternal postnatal anxiety symptoms |
| #1287 | 1 | maternal postpartum depression |
| #1288 | 1 | maternal prenatal anxiety symptoms |
| #1289 | 1 | mcgurk illusion |
| #1290 | 1 | meaningfulness |
| #1291 | 1 | media exposure |
| #1292 | 1 | mediated priming |
| #1293 | 1 | mediation |
| #1294 | 1 | medically underserved area |
| #1295 | 1 | memory development |
| #1296 | 1 | mental development |
| #1297 | 1 | mental disease |
| #1298 | 1 | mental function |
| #1299 | 1 | mental health |
| #1300 | 1 | mental performance |
| #1301 | 1 | mental representation |
| #1302 | 1 | mental task |
| #1303 | 1 | mentalization |
| #1304 | 1 | method comparison |
| #1305 | 1 | metronidazole |
| #1306 | 1 | mimicry (biology) |
| #1307 | 1 | mind |
| #1308 | 1 | mini-grammars |
| #1309 | 1 | minimal pairs |
| #1310 | 1 | mirror test |
| #1311 | 1 | mismatch |
| #1312 | 1 | mobile applications |
| #1313 | 1 | model update |
| #1314 | 1 | moderate prematurity |
| #1315 | 1 | monitoring |
| #1316 | 1 | monkey faces |
| #1317 | 1 | monocular vision |
| #1318 | 1 | monte carlo method |
| #1319 | 1 | moral cognition |
| #1320 | 1 | moral development |
| #1321 | 1 | morals |
| #1322 | 1 | motion parallax |
| #1323 | 1 | motor |
| #1324 | 1 | motor control |
| #1325 | 1 | motor experience |
| #1326 | 1 | motor learning |
| #1327 | 1 | motor resonance |
| #1328 | 1 | motor resonance account |
| #1329 | 1 | mouse |
| #1330 | 1 | mouth (anatomy) |
| #1331 | 1 | movement representations |
| #1332 | 1 | moving target vs background texture & monocular vs binocular viewing |
| #1333 | 1 | mpfc |
| #1334 | 1 | mri |
| #1335 | 1 | msel |
| #1336 | 1 | multi-word sequences |
| #1337 | 1 | multimodal behaviors |
| #1338 | 1 | multimodal labels |
| #1339 | 1 | multimodal learning |
| #1340 | 1 | multiple identity tracking |
| #1341 | 1 | multiple object tracking |
| #1342 | 1 | multiracial |
| #1343 | 1 | multiscale entropy |
| #1344 | 1 | multisensory |
| #1345 | 1 | multisensory perceptual narrowing |
| #1346 | 1 | multisensory processing |
| #1347 | 1 | mutual gaze |
| #1348 | 1 | nap |
| #1349 | 1 | natural language processing |
| #1350 | 1 | natural language processing systems |
| #1351 | 1 | naturalistic |
| #1352 | 1 | neck |
| #1353 | 1 | negative affect |
| #1354 | 1 | negative emotions |
| #1355 | 1 | neonatal |
| #1356 | 1 | nerve fibers |
| #1357 | 1 | nervous system function |
| #1358 | 1 | neural networks |
| #1359 | 1 | neural networks (computer) |
| #1360 | 1 | neurodevelopmental outcome |
| #1361 | 1 | neurologic disease |
| #1362 | 1 | neurological |
| #1363 | 1 | neurological damage |
| #1364 | 1 | neurophysiology |
| #1365 | 1 | neuropsychological test |
| #1366 | 1 | neurovascular coupling |
| #1367 | 1 | newborns |
| #1368 | 1 | nicotinic |
| #1369 | 1 | nitrogen fixation |
| #1370 | 1 | noise |
| #1371 | 1 | noisy input |
| #1372 | 1 | non-linguistic representations |
| #1373 | 1 | non-native phonemes |
| #1374 | 1 | non-social cues |
| #1375 | 1 | non-symbolic |
| #1376 | 1 | non-verbal ability |
| #1377 | 1 | non-weird psychology |
| #1378 | 1 | nonhuman primate |
| #1379 | 1 | nonparametric |
| #1380 | 1 | nonverbal |
| #1381 | 1 | normal distribution |
| #1382 | 1 | note |
| #1383 | 1 | noun learning |
| #1384 | 1 | novelty preference |
| #1385 | 1 | number |
| #1386 | 1 | number discrimination |
| #1387 | 1 | nutrition |
| #1388 | 1 | nutritional status |
| #1389 | 1 | object categorization |
| #1390 | 1 | object choice |
| #1391 | 1 | object expectations |
| #1392 | 1 | object files |
| #1393 | 1 | object individuation |
| #1394 | 1 | object labelling |
| #1395 | 1 | object location |
| #1396 | 1 | object occlusion |
| #1397 | 1 | object perception |
| #1398 | 1 | object perception development |
| #1399 | 1 | object perception in infancy |
| #1400 | 1 | object permanence |
| #1401 | 1 | object persistence |
| #1402 | 1 | object prehension |
| #1403 | 1 | object representation |
| #1404 | 1 | object size |
| #1405 | 1 | object solidity |
| #1406 | 1 | object-based attention |
| #1407 | 1 | object-directed actions |
| #1408 | 1 | object-processing |
| #1409 | 1 | observational study |
| #1410 | 1 | obstetric delivery |
| #1411 | 1 | occluded trajectory |
| #1412 | 1 | ocular accommodation |
| #1413 | 1 | ocular physiological phenomena |
| #1414 | 1 | oculomotor paradigm |
| #1415 | 1 | odorants |
| #1416 | 1 | online language processing |
| #1417 | 1 | online research |
| #1418 | 1 | onomatopoeia |
| #1419 | 1 | open data |
| #1420 | 1 | optokinetic nystagmus |
| #1421 | 1 | optokinetic reflex & nystagmus |
| #1422 | 1 | orienting attention |
| #1423 | 1 | ostension |
| #1424 | 1 | outcome prediction |
| #1425 | 1 | overt attention |
| #1426 | 1 | overt orienting response |
| #1427 | 1 | ovum |
| #1428 | 1 | own-race |
| #1429 | 1 | oxtr epigenetics |
| #1430 | 1 | p.h.s. |
| #1431 | 1 | paediatric neurology |
| #1432 | 1 | pan troglodytes |
| #1433 | 1 | pantomime |
| #1434 | 1 | parameters |
| #1435 | 1 | parent-child interaction |
| #1436 | 1 | parent-infant interaction |
| #1437 | 1 | parental anxiety |
| #1438 | 1 | parental care |
| #1439 | 1 | parental value transmission |
| #1440 | 1 | parenting hassles |
| #1441 | 1 | parent‐reported symptoms |
| #1442 | 1 | parent–child interaction |
| #1443 | 1 | parent–child play |
| #1444 | 1 | parsing |
| #1445 | 1 | passive movement |
| #1446 | 1 | path obstruction |
| #1447 | 1 | pathologic |
| #1448 | 1 | patient preference |
| #1449 | 1 | pca |
| #1450 | 1 | peer interaction |
| #1451 | 1 | perceived stress |
| #1452 | 1 | perception action coupling |
| #1453 | 1 | perception and action |
| #1454 | 1 | perception-action |
| #1455 | 1 | perception–action |
| #1456 | 1 | perceptual attunement |
| #1457 | 1 | perceptual continua |
| #1458 | 1 | perceptual disorders |
| #1459 | 1 | perceptual information |
| #1460 | 1 | perceptual learning |
| #1461 | 1 | perceptual motor coordination |
| #1462 | 1 | perceptual motor processes |
| #1463 | 1 | performance rankings |
| #1464 | 1 | perimetry |
| #1465 | 1 | perinatal risk factors |
| #1466 | 1 | persistence |
| #1467 | 1 | personality development |
| #1468 | 1 | personalized medicine |
| #1469 | 1 | pets |
| #1470 | 1 | pharmacology |
| #1471 | 1 | phonetic cues |
| #1472 | 1 | phonological |
| #1473 | 1 | phonological bias |
| #1474 | 1 | phonology and phonetics of stress |
| #1475 | 1 | photoreceptor |
| #1476 | 1 | phrasal prosody |
| #1477 | 1 | phthalates |
| #1478 | 1 | phthalic acids |
| #1479 | 1 | physical salience |
| #1480 | 1 | physiologic |
| #1481 | 1 | physiological arousal |
| #1482 | 1 | physiological feedback |
| #1483 | 1 | pictorial depth cues |
| #1484 | 1 | pilot projects |
| #1485 | 1 | planning |
| #1486 | 1 | plasticity |
| #1487 | 1 | pointing cues |
| #1488 | 1 | polygenic score |
| #1489 | 1 | polymorphism |
| #1490 | 1 | postnatal depression |
| #1491 | 1 | postpartum |
| #1492 | 1 | postpartum depression |
| #1493 | 1 | pre |
| #1494 | 1 | predication |
| #1495 | 1 | prediction accuracy |
| #1496 | 1 | predictive eye movements |
| #1497 | 1 | predictive eye-movements |
| #1498 | 1 | predictive gaze |
| #1499 | 1 | predictive value |
| #1500 | 1 | preferential looking paradigm |
| #1501 | 1 | preferential reaching paradigm |
| #1502 | 1 | prenatal care |
| #1503 | 1 | prenatal exposure |
| #1504 | 1 | prenatal glucocorticoid-exposure |
| #1505 | 1 | presbyopia |
| #1506 | 1 | preterm infant |
| #1507 | 1 | primary health care |
| #1508 | 1 | primates |
| #1509 | 1 | principal component analysis |
| #1510 | 1 | probability intuitions in infancy |
| #1511 | 1 | procedural knowledge |
| #1512 | 1 | procedural memory |
| #1513 | 1 | procedures |
| #1514 | 1 | processing |
| #1515 | 1 | processing of same-and other-race faces |
| #1516 | 1 | prodromal intervention |
| #1517 | 1 | productive verbs |
| #1518 | 1 | productive vocabulary |
| #1519 | 1 | prognosis |
| #1520 | 1 | program evaluation |
| #1521 | 1 | progressive alignment |
| #1522 | 1 | pronunciation |
| #1523 | 1 | prosocial behaviour |
| #1524 | 1 | prosodic cues |
| #1525 | 1 | prosody |
| #1526 | 1 | prospective control |
| #1527 | 1 | pseudoneglect |
| #1528 | 1 | pseudowords |
| #1529 | 1 | psychiatric status rating scales |
| #1530 | 1 | psychological development |
| #1531 | 1 | psychological distance |
| #1532 | 1 | psychological engagement |
| #1533 | 1 | psychological theory |
| #1534 | 1 | psychosocial development |
| #1535 | 1 | pupil (eye) |
| #1536 | 1 | pupillary light reflex |
| #1537 | 1 | pyramidal tracts |
| #1538 | 1 | qualitative differences |
| #1539 | 1 | race (anthropological) |
| #1540 | 1 | racial and ethnic differences |
| #1541 | 1 | racial bias |
| #1542 | 1 | racism |
| #1543 | 1 | randomized controlled trial |
| #1544 | 1 | rational action understanding |
| #1545 | 1 | reactive inhibition |
| #1546 | 1 | reading |
| #1547 | 1 | reading development |
| #1548 | 1 | real-time |
| #1549 | 1 | real-time processing |
| #1550 | 1 | reality |
| #1551 | 1 | reality testing |
| #1552 | 1 | reasoned action |
| #1553 | 1 | reasoning |
| #1554 | 1 | receptive vocabulary |
| #1555 | 1 | receptors |
| #1556 | 1 | reciprocity |
| #1557 | 1 | recreation |
| #1558 | 1 | recurrence |
| #1559 | 1 | recurrence quantification analysis |
| #1560 | 1 | recurrence quantification analysis (rqa) |
| #1561 | 1 | recurrent disease |
| #1562 | 1 | reference |
| #1563 | 1 | referential expectation |
| #1564 | 1 | reflexes |
| #1565 | 1 | refraction |
| #1566 | 1 | regularities |
| #1567 | 1 | rem sleep |
| #1568 | 1 | representational gestures |
| #1569 | 1 | representations |
| #1570 | 1 | research design |
| #1571 | 1 | resource allocation |
| #1572 | 1 | respiratory distress syndrome |
| #1573 | 1 | responding joint attention |
| #1574 | 1 | response duration |
| #1575 | 1 | response latency |
| #1576 | 1 | response time |
| #1577 | 1 | reticular formation |
| #1578 | 1 | retinal locus of regard & consistency of refixations |
| #1579 | 1 | retrolental fibroplasia |
| #1580 | 1 | reward learning |
| #1581 | 1 | reward processing |
| #1582 | 1 | right hemisphere |
| #1583 | 1 | robot gaze |
| #1584 | 1 | robot’s face |
| #1585 | 1 | roc curve |
| #1586 | 1 | romance languages |
| #1587 | 1 | rural population |
| #1588 | 1 | saccade directions |
| #1589 | 1 | saccade toward faces |
| #1590 | 1 | saccadic control |
| #1591 | 1 | saccadic eye movements |
| #1592 | 1 | saccadic reaction time |
| #1593 | 1 | saccadic responses |
| #1594 | 1 | sadness |
| #1595 | 1 | salience |
| #1596 | 1 | saliency map |
| #1597 | 1 | saliency models |
| #1598 | 1 | saliva |
| #1599 | 1 | same-race preferences |
| #1600 | 1 | same-rhythm-class languages |
| #1601 | 1 | scale error |
| #1602 | 1 | scale errors |
| #1603 | 1 | scanning |
| #1604 | 1 | scanning strategies |
| #1605 | 1 | scenes |
| #1606 | 1 | scientific replication |
| #1607 | 1 | screening methods |
| #1608 | 1 | selective imitation |
| #1609 | 1 | selective vs. exact imitation |
| #1610 | 1 | self report |
| #1611 | 1 | self-locomotion |
| #1612 | 1 | self-produced locomotion |
| #1613 | 1 | semantic knowledge |
| #1614 | 1 | semantic priming |
| #1615 | 1 | sensitive periods |
| #1616 | 1 | sensitivity to ungrammaticality |
| #1617 | 1 | sensori-motor adaptation |
| #1618 | 1 | sensorimotor coordination |
| #1619 | 1 | sensorimotor development |
| #1620 | 1 | sensorimotor simulation |
| #1621 | 1 | sensory |
| #1622 | 1 | sensory thresholds |
| #1623 | 1 | sentence processing |
| #1624 | 1 | sequential action |
| #1625 | 1 | sequential learning |
| #1626 | 1 | serial learning |
| #1627 | 1 | serial reaction time task |
| #1628 | 1 | service animals |
| #1629 | 1 | set shifting |
| #1630 | 1 | sex and race categorization |
| #1631 | 1 | sex perception in infancy |
| #1632 | 1 | sexually dimorphic |
| #1633 | 1 | shared attention |
| #1634 | 1 | shared signal hypothesis |
| #1635 | 1 | short-term memory |
| #1636 | 1 | shyness |
| #1637 | 1 | sierra leone |
| #1638 | 1 | signal processing |
| #1639 | 1 | silence |
| #1640 | 1 | silently talking faces |
| #1641 | 1 | simulation |
| #1642 | 1 | singing |
| #1643 | 1 | single-blind method |
| #1644 | 1 | size |
| #1645 | 1 | sleep disorder |
| #1646 | 1 | sleep duration |
| #1647 | 1 | sleep-dependent memory consolidation |
| #1648 | 1 | small for date infant |
| #1649 | 1 | small forms |
| #1650 | 1 | small-scale societies |
| #1651 | 1 | smooth pursuit eye movements in single target tracking & optokinetic nystagmus |
| #1652 | 1 | social ability |
| #1653 | 1 | social aspect |
| #1654 | 1 | social behavior disorders |
| #1655 | 1 | social cognition and social behavior |
| #1656 | 1 | social decision-making |
| #1657 | 1 | social engagement |
| #1658 | 1 | social exclusion |
| #1659 | 1 | social fear |
| #1660 | 1 | social information processing |
| #1661 | 1 | social motivation |
| #1662 | 1 | social participation |
| #1663 | 1 | social preferences |
| #1664 | 1 | social responsiveness |
| #1665 | 1 | social status |
| #1666 | 1 | social visual engagement |
| #1667 | 1 | social-communicative cues |
| #1668 | 1 | social-pragmatic attention |
| #1669 | 1 | socio-economic status |
| #1670 | 1 | sociodemographic factors |
| #1671 | 1 | sound cues |
| #1672 | 1 | sound localization |
| #1673 | 1 | sound symbolism |
| #1674 | 1 | south africa |
| #1675 | 1 | spatial asymmetry |
| #1676 | 1 | spatial cueing |
| #1677 | 1 | spatial discrimination |
| #1678 | 1 | spatial language |
| #1679 | 1 | spatial learning |
| #1680 | 1 | spatial memory |
| #1681 | 1 | spatial object processing |
| #1682 | 1 | spatial orientation (perception) |
| #1683 | 1 | spatial proximity |
| #1684 | 1 | spatial relational memory |
| #1685 | 1 | spatial transformation |
| #1686 | 1 | spatio-temporal analysis |
| #1687 | 1 | spatiotemporal analysis |
| #1688 | 1 | species recognition |
| #1689 | 1 | species specific |
| #1690 | 1 | species specificity |
| #1691 | 1 | speech acoustics |
| #1692 | 1 | speech cues |
| #1693 | 1 | speech discrimination |
| #1694 | 1 | speech disorders |
| #1695 | 1 | speech intelligibility |
| #1696 | 1 | speech learning |
| #1697 | 1 | speech preference |
| #1698 | 1 | speech stream |
| #1699 | 1 | speech variability |
| #1700 | 1 | speech-gesture synchrony |
| #1701 | 1 | speed-accuracy trade-off |
| #1702 | 1 | spoken word comprehension |
| #1703 | 1 | spontaneous monitoring |
| #1704 | 1 | spreading activation |
| #1705 | 1 | starting points |
| #1706 | 1 | static real‐world stimuli |
| #1707 | 1 | static vidio images |
| #1708 | 1 | statistical word segmentation |
| #1709 | 1 | stereoscopic vision |
| #1710 | 1 | steroid |
| #1711 | 1 | sticky mittens |
| #1712 | 1 | stimuli |
| #1713 | 1 | stimulus anticipation |
| #1714 | 1 | stimulus change |
| #1715 | 1 | stimulus complexity |
| #1716 | 1 | stimulus duration |
| #1717 | 1 | stimulus onset |
| #1718 | 1 | stimulus parameters |
| #1719 | 1 | stimulus properties |
| #1720 | 1 | storyline |
| #1721 | 1 | storylines |
| #1722 | 1 | stranger anxiety |
| #1723 | 1 | stress |
| #1724 | 1 | structural connectivity |
| #1725 | 1 | structure-from-motion |
| #1726 | 1 | subliminal processing |
| #1727 | 1 | submentalizing |
| #1728 | 1 | subtraction |
| #1729 | 1 | sucking behavior |
| #1730 | 1 | superior colliculi |
| #1731 | 1 | surgical instruments |
| #1732 | 1 | surgical training |
| #1733 | 1 | surprise |
| #1734 | 1 | sustained attention |
| #1735 | 1 | sustained visual attention |
| #1736 | 1 | syllable discrimination |
| #1737 | 1 | symbolism |
| #1738 | 1 | symptoms |
| #1739 | 1 | synchrony perception |
| #1740 | 1 | syntactic ambiguity resolution |
| #1741 | 1 | syntactic bootstrapping |
| #1742 | 1 | syntactic representations |
| #1743 | 1 | syntax |
| #1744 | 1 | task |
| #1745 | 1 | task-evoked pupil response |
| #1746 | 1 | task-evoked pupil responses |
| #1747 | 1 | teleological inferences |
| #1748 | 1 | teleological reasoning |
| #1749 | 1 | television |
| #1750 | 1 | telicity |
| #1751 | 1 | temperamental characteristics |
| #1752 | 1 | temperamental shyness |
| #1753 | 1 | temporal asynchrony |
| #1754 | 1 | tendency to follow speaker's attentional shift to object |
| #1755 | 1 | term birth |
| #1756 | 1 | test bias |
| #1757 | 1 | testosterone |
| #1758 | 1 | texture perception |
| #1759 | 1 | third-party interaction |
| #1760 | 1 | third-person evaluation |
| #1761 | 1 | threat detection |
| #1762 | 1 | threat processing |
| #1763 | 1 | time factor |
| #1764 | 1 | time perception |
| #1765 | 1 | tocopherols |
| #1766 | 1 | toddlerhood |
| #1767 | 1 | tonic pupil |
| #1768 | 1 | top– down |
| #1769 | 1 | touch |
| #1770 | 1 | touch perception |
| #1771 | 1 | tova |
| #1772 | 1 | toy preferences |
| #1773 | 1 | training |
| #1774 | 1 | transient features |
| #1775 | 1 | transition probabilities |
| #1776 | 1 | transitive structures |
| #1777 | 1 | treatment outcome |
| #1778 | 1 | trust |
| #1779 | 1 | twins |
| #1780 | 1 | typically developing |
| #1781 | 1 | u.s. gov't |
| #1782 | 1 | uganda |
| #1783 | 1 | uncanny valley |
| #1784 | 1 | understanding others' needs |
| #1785 | 1 | united states |
| #1786 | 1 | utate |
| #1787 | 1 | validity |
| #1788 | 1 | vanuatu |
| #1789 | 1 | velocity |
| #1790 | 1 | verbal behavior |
| #1791 | 1 | verbal comprehension |
| #1792 | 1 | verbal memory |
| #1793 | 1 | verbal shadowing |
| #1794 | 1 | vertical gaze |
| #1795 | 1 | very preterm |
| #1796 | 1 | video deficit effect |
| #1797 | 1 | videorecording |
| #1798 | 1 | videotape recording |
| #1799 | 1 | vigilance |
| #1800 | 1 | violation of expectation |
| #1801 | 1 | virtual agent |
| #1802 | 1 | vision test |
| #1803 | 1 | visual and auditory perceptual salience |
| #1804 | 1 | visual anticipation |
| #1805 | 1 | visual array task (vat) |
| #1806 | 1 | visual behavior |
| #1807 | 1 | visual cueing |
| #1808 | 1 | visual cues |
| #1809 | 1 | visual disengagement |
| #1810 | 1 | visual evoked potential |
| #1811 | 1 | visual fixation |
| #1812 | 1 | visual fixation trajectories |
| #1813 | 1 | visual form perception |
| #1814 | 1 | visual illusion |
| #1815 | 1 | visual information |
| #1816 | 1 | visual paired comparison |
| #1817 | 1 | visual paired-comparison |
| #1818 | 1 | visual perspective taking |
| #1819 | 1 | visual prediction |
| #1820 | 1 | visual preferences |
| #1821 | 1 | visual processing |
| #1822 | 1 | visual recognition memory |
| #1823 | 1 | visual representations |
| #1824 | 1 | visual responses |
| #1825 | 1 | visual social attention |
| #1826 | 1 | visual speech cues |
| #1827 | 1 | visual stm |
| #1828 | 1 | visual world |
| #1829 | 1 | visual world paradigm |
| #1830 | 1 | visually guided behavior |
| #1831 | 1 | visuo-manual coordination |
| #1832 | 1 | visuo-spatial functions |
| #1833 | 1 | visuo-spatial orienting |
| #1834 | 1 | visuospatial attention orienting |
| #1835 | 1 | vocabulary acquisition |
| #1836 | 1 | vocabulary skills |
| #1837 | 1 | vocal imitation |
| #1838 | 1 | vocalizations |
| #1839 | 1 | voice quality |
| #1840 | 1 | vpc |
| #1841 | 1 | waist-to-hip ratio |
| #1842 | 1 | war |
| #1843 | 1 | weight |
| #1844 | 1 | weight perception |
| #1845 | 1 | within-person variability |
| #1846 | 1 | word frequency |
| #1847 | 1 | word-object mapping |
| #1848 | 1 | younger siblings |
| #1849 | 1 | zika |
| #1850 | 1 | zika virus |
| #1851 | 1 | zika virus infection |
